# Supplementary material for: Interventional hydrogel microsphere vaccine as an immune amplifier for activated antitumour immunity after ablation therapy
Source: Nat Commun. 2023 Jul 11;14:4106. doi: 10.1038/s41467-023-39759-w (PMC10336067; doi:10.1038/s41467-023-39759-w)
Supplement: Supplementary file 1 — Supplementary Information [file 41467_2023_39759_MOESM1_ESM.pdf]

Supplementary Information

Interventional hydrogel microsphere vaccine as an immune amplifier for activated antitumour immunity after ablation therapy

Xiaoyu Liu, Yaping Zhuang, Wei Huang, Zhuozhuo Wu, Yingjie Chen, Qungang Shan, Yuefang Zhang, Zhiyuan Wu, Xiaoyi Ding, Zilong Qiu, Wenguo Cui, Zhongmin Wang

Supplementary Inventory

Supplementary Fig. 1. The physicochemical characterization data of hydrogel microspheres.

Supplementary Fig. 2. The characterization of the pH dependence of CD40L release and the concentration of Cas9 plasmid in the hydrogel system.

Supplementary Fig. 3. Flow cytometry gating strategy for the analysis of BMDC maturation and activation in vitro.

Supplementary Fig. 4. The proliferation and differentiation of bone marrow derived cells towards cDC1s and macrophages.

Supplementary Fig. 5. Representative images of CK19- and αSMA-labelled immunofluorescence staining of pancreas in *Kras*$^{LSL\text{-}G12D}$; *Tp53*$^{fl/-}$; *Pdx1*-Cre (KPC) mouse and mouse bearing KPC orthotopic pancreatic tumour.

Supplementary Fig. 6. Measurement of the intratumour pH value.

Supplementary Fig. 7. Design of the CRISPR gRNAs targeting mouse CD274 gene.

Supplementary Fig. 8. Quantification of the Western blotting.

Supplementary Fig. 9. Analysis the tumour necrosis, CD8$^+$ and CD103$^+$ immune cell infiltration across different treatment groups.

Supplementary Fig. 10. Flow cytometry gating strategy for the analysis of tumour-resident DCs.

Supplementary Fig. 11. Flow cytometry gating strategy for the analysis of DCs migrated into TdLNs and spleens.

Supplementary Fig. 12. Flow cytometry gating strategy for the analysis of OVA$_{257\text{-}246}$-H-2Kb-specific tumour-resident cDC1s.

Supplementary Fig. 13. Flow cytometry gating strategy for the analysis of the intracellular expression of Ki67, IFN-γ and TNF-α in T cells.

Supplementary Fig. 14. Representative images of CD8- and CD4-labelled immunofluorescence staining of pancreas lymph nodes (pLNs) in tumour bearing mice.

Supplementary Fig. 15. The density of cDC1s was correlated with the density of activated CD8$^+$ T cells in TdLNs.

Supplementary Fig. 16. Flow cytometry gating strategy for the analysis of (a) tumour-infiltrating Ly6G$^+$CD11b$^+$ myeloid cells and NK1.1$^+$ cells and (b) quantification analysis.

Supplementary Fig. 17. Flow cytometry gating strategy for immune phenotyping of the macrophages.

Supplementary Fig. 18. Flow cytometry gating strategy for the analysis of tumour-infiltrating $FoxP3^{+}CD25^{+}$ Tregs, $Ki67^{+}CD8^{+}$ T cells, and IFN-$\gamma^{+}CD8^{+}$ T cells.

Supplementary Fig. 19. Representative H&E staining images of major organs in KPC orthotopic tumour bearing mice receiving different therapies.

Supplementary Fig. 20. The efficacy of intravenous delivery of αCD40 and FLT3L against KPC tumour.

Supplementary Fig. 21. Flow cytometry gating strategy for the analysis of $CD8^{+}$ T cells, $CD4^{+}$ T cells, $CD24^{+}$MHC II^{+}DC-like cells and $CD103^{+}$ cDC1-like cells in circulation.

Supplementary Fig. 22. Representative images show the gross distant metastasis tumours across different groups.

Supplementary Fig. 23. Western Blot assay demonstrating the expression level molecular markers in KPC and Panc02 cells, and the expression of PD-L1 in IFN-γ treatment Panc02 cells.

Supplementary Table 1. Antibody information.

Supplementary Fig. 1

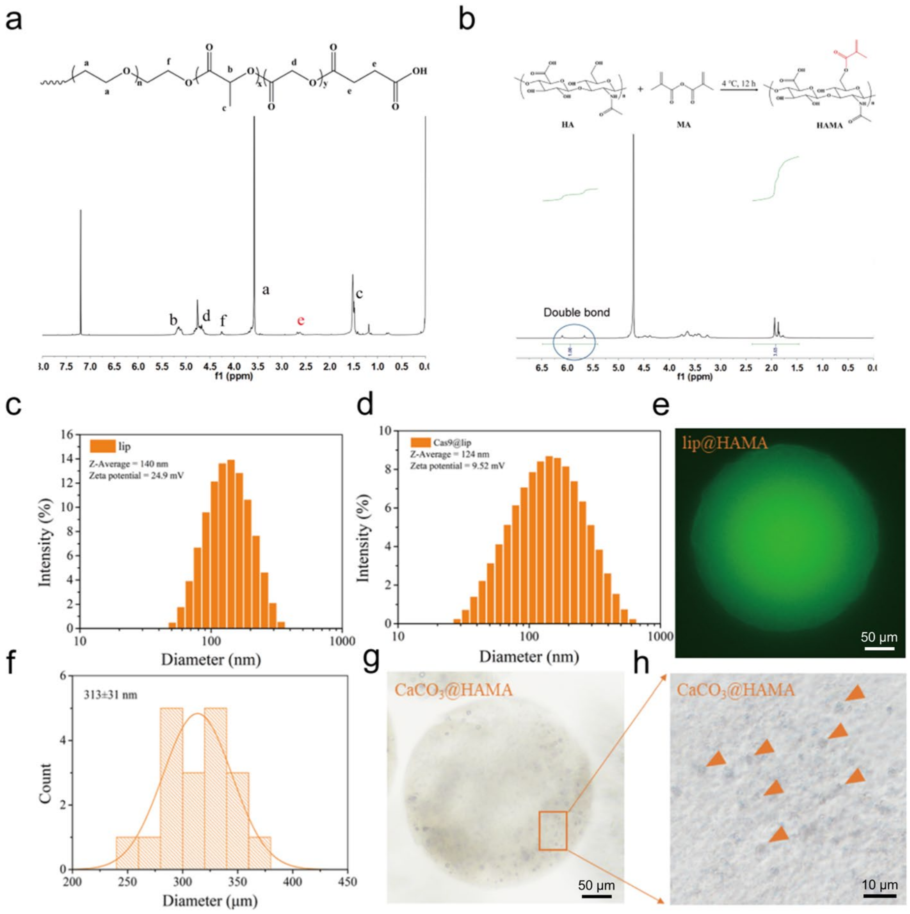

Supplementary Fig. 1. The physicochemical characterization data of hydrogel microspheres. (a) The ^{1}H nuclear magnetic resonance (^{1}H NMR) spectrum of the terminal modified PLGA-PEG-PLGA, the signal peak in the nuclear magnetic spectrum (^{1}H NMR) at a chemical shift of 2.68 ppm attributable to methylene protons ($-CH_2CH_2-$) of the succinyl unit. **(b)** The ^{1}H nuclear magnetic resonance (^{1}H NMR) spectrum of HAMA, the signal peaks at 5.66 and 6.10 PPM were attributable to methacrylate anhydride's double bond hydrogen, with a double bond modification rate of 69.8%. (**c**) Hydrodynamic diameter of the lip; (**d**) Hydrodynamic diameter and zeta potential of the Cas9@lip; (**e**) Fluorescence photograph of FITC@lip/HAMA hydrogel microspheres; (**f**) The particle size statistics of hydrogel microspheres by image J; (**g**) The photograph of $CaCO_3$@HAMA hydrogel microspheres; (**h**) The enlarged image of $CaCO_3$@HAMA hydrogel microspheres. Source data are provided as a Source data file.

Supplementary Fig. 2

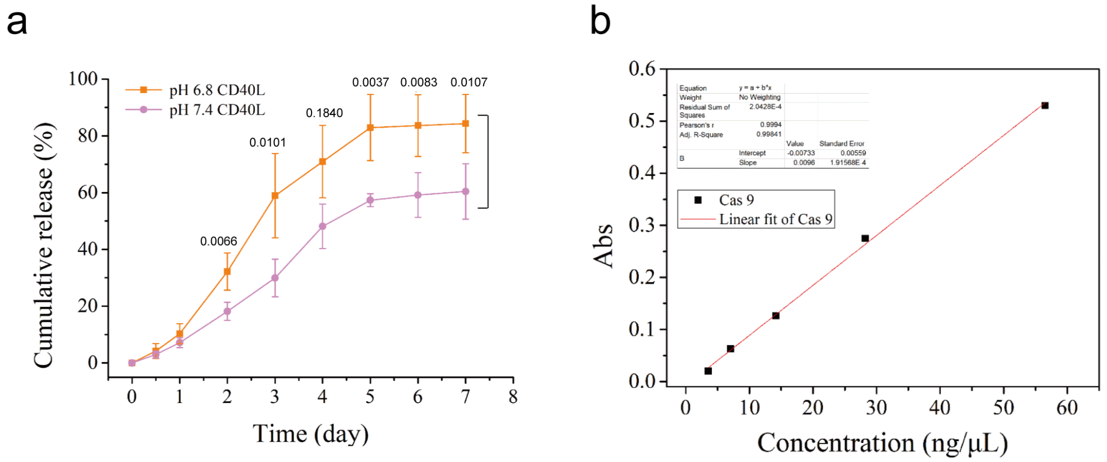

Supplementary Fig. 2. The characterization of the pH dependence of CD40L release and the concentration of Cas9 plasmid in the hydrogel system. (a) The release of CD40L from CD40L@$CaCO_3$ /Flt3L/Cas9@lip/HAMA hydrogel microsphere at pH value of 6.8 and 7.4 in 1×PBS solution. n=3 independent experiments. Data are presented as mean values +/- SD. Two-tailed unpaired t-test. **(b)** The standard curve of Cas9 plasmid DNA according to UV absorption analysis. Data are presented as linear regression. Source data are provided as a Source data file.

Supplementary Fig. 3

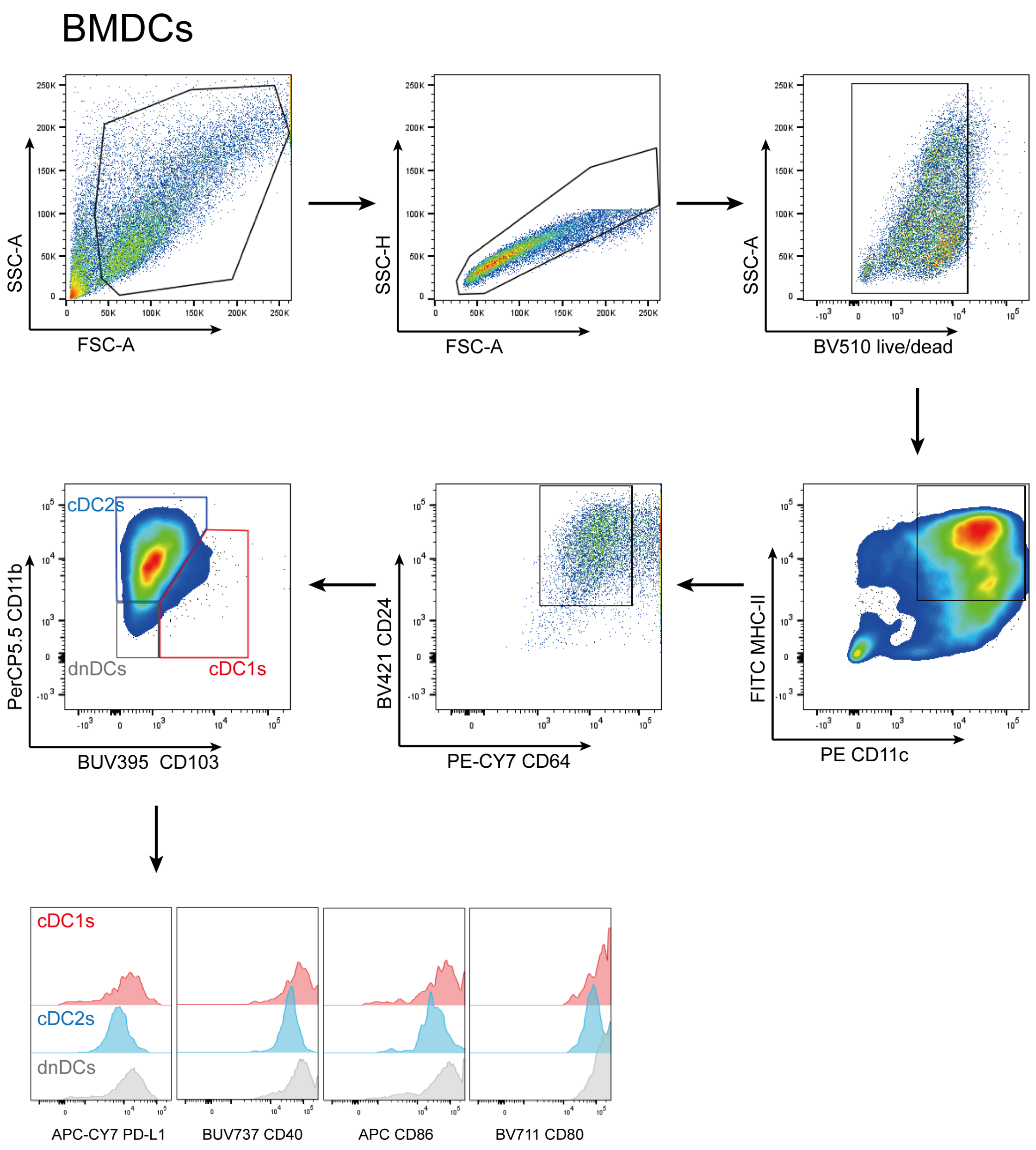

Supplementary Fig. 3. Flow cytometry gating strategy for the analysis of BMDC maturation and activation *in vitro*. The gating strategy identifies $CD103^{+}$ $CD11b^{-}$ cDC1s, $CD103^{-}CD11b^{+}$ cDC2s, $CD103^{-}CD11b^{-}$ DCs (dnDCs) and the level of costimulators (Fig. 2q).

Supplementary Fig. 4

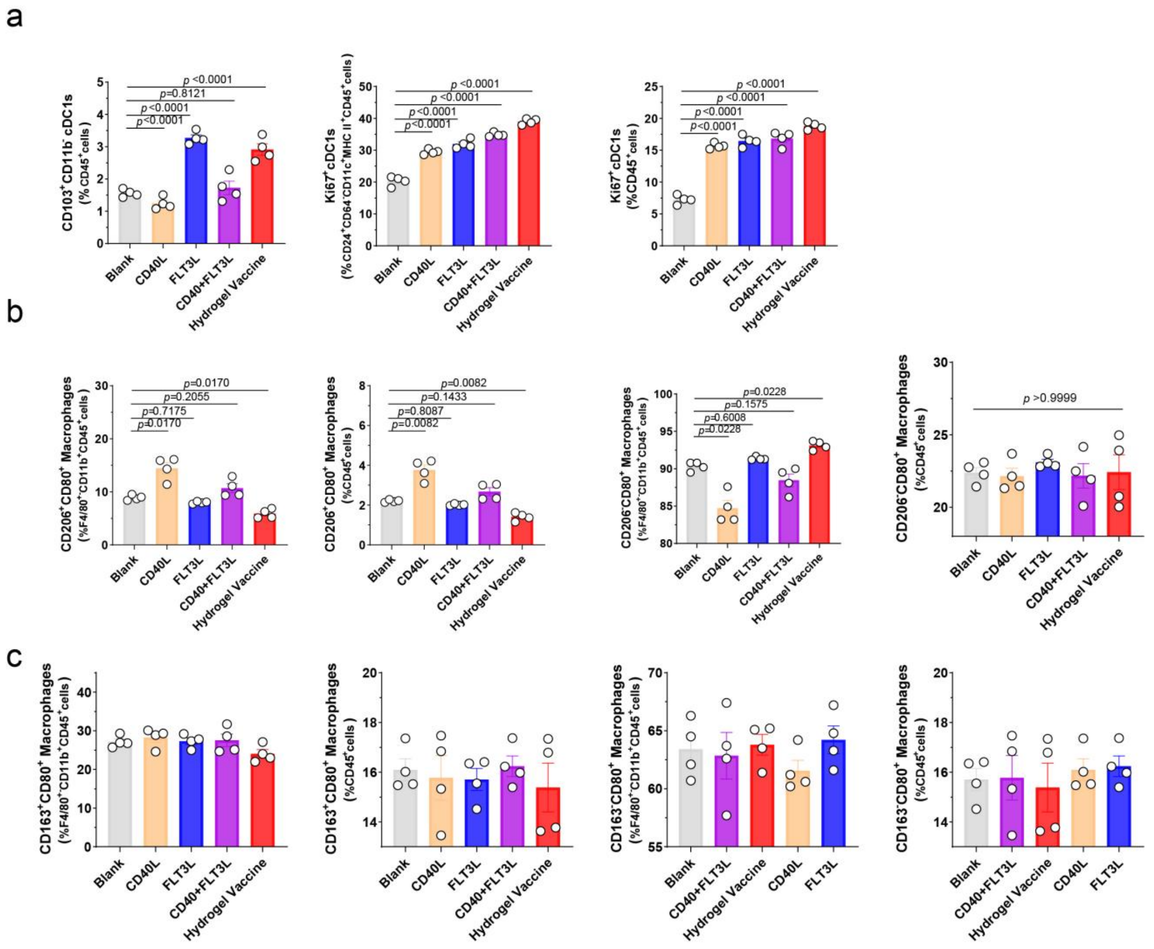

Supplementary Fig. 4. The proliferation and differentiation of bone-marrow derived cells towards cDC1s and macrophages. (**a**) Analysis of the effect of hydrogel microsphere vaccines and the counterparts on the differentiation and proliferation in cDC1s; (**b-c**) Analysis of the effect of hydrogel microsphere vaccines and the counterparts on the differentiation of BMDC into macrophages. The $CD163^{+}CD80^{+}$ macrophages and $CD206^{+}CD80^{+}$ macrophages represented the M2-like macrophages. The $CD163^{-}CD80^{+}$ macrophages and $CD163^{-}CD80^{+}$ macrophages represented the M1-like macrophages. Fore **a-c**, n=4 biologically independent samples. data are presented as mean values +/- SEM. Two-tailed Dunnett t-test. Source data are provided as a Source data file.

Supplementary Fig. 5

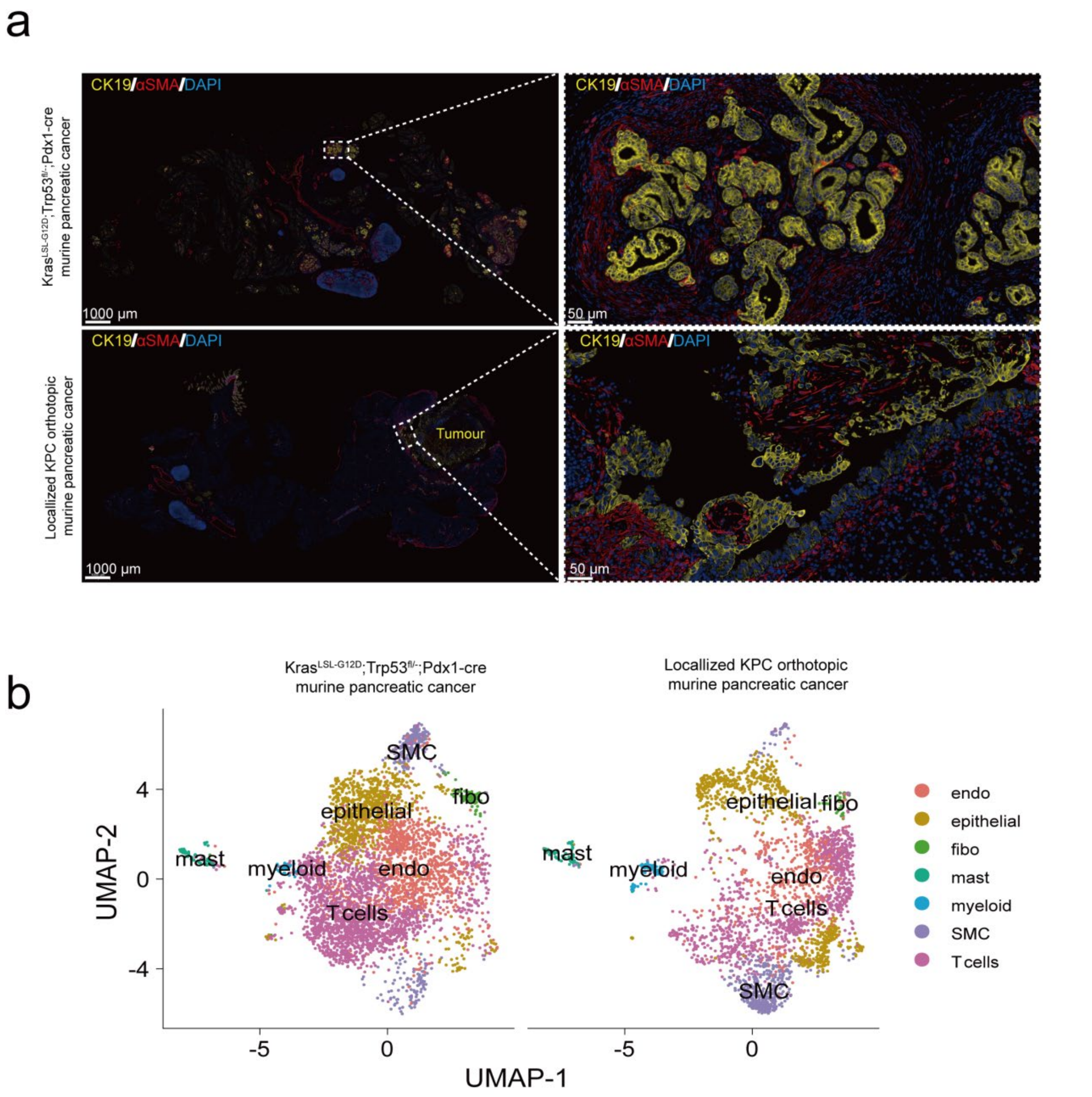

Supplementary Fig. 5. Representative images of CK19- and αSMA-labelled immunofluorescence staining of pancreas in Kras$^{LSL-G12D}$; Tp53$^{fl/-}$; Pdx1-Cre (KPC) mouse and mouse bearing KPC orthotopic pancreatic tumour. (**a**) Representative images of CK19- and αSMA-labelled immunofluorescence staining. 3 biologically independent samples were performed to confirm data stability. (**b**) Single-cells analysis demonstrated the tumour-infiltrating immune cells of pancreatic cancers in KPC genetic mouse and mouse bearing KPC orthotopic pancreatic tumours. Source data are provided as a Source data file.

Supplementary Fig. 6

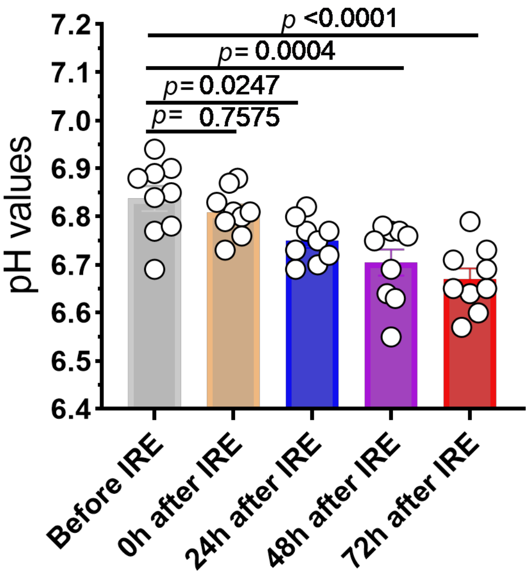

Supplementary Fig. 6. Measurement of the intratumour pH value. The intratumour pH value before and after the IRE therapy. There were three mice in each group, and each tumour was tested for three times. n=9 biologically independent samples. data are presented as mean values +/- SEM. Two-tailed Dunnett t-test. Source data are provided as a Source data file.

Supplementary Fig. 7

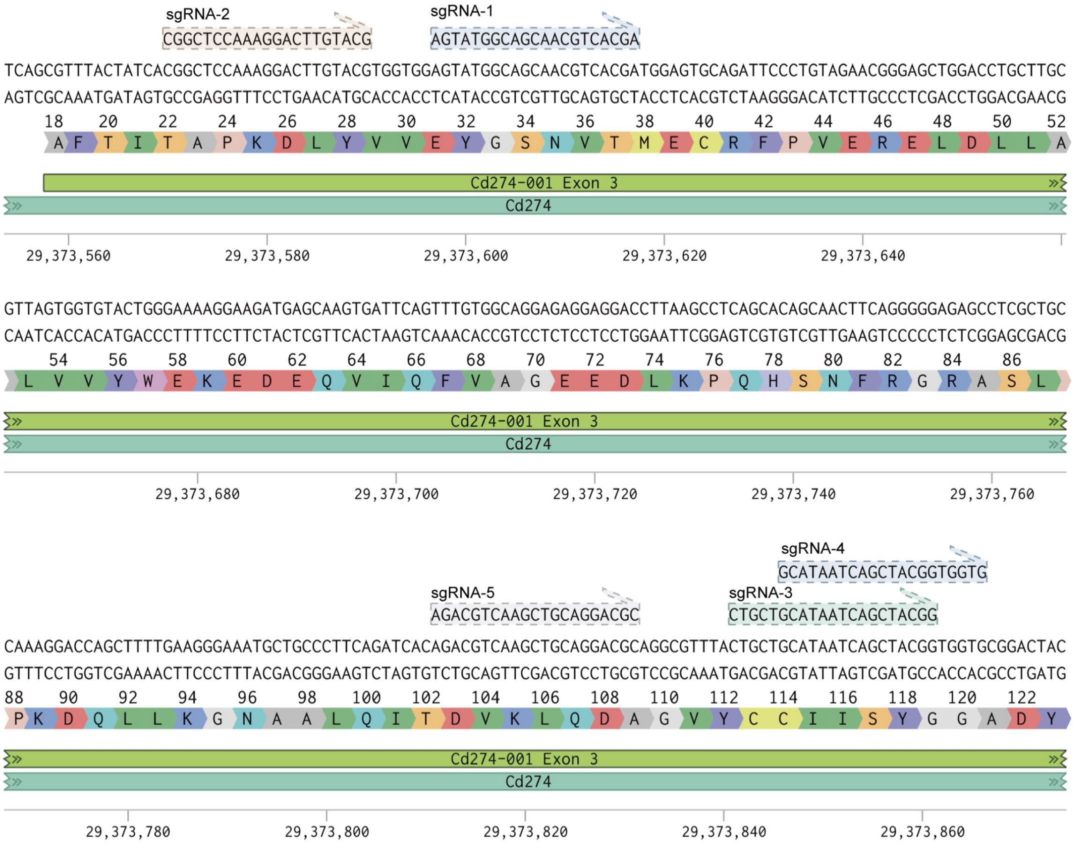

Supplementary Fig. 7. Design of the CRISPR gRNAs targeting mouse CD274 gene.

Supplementary Fig. 8

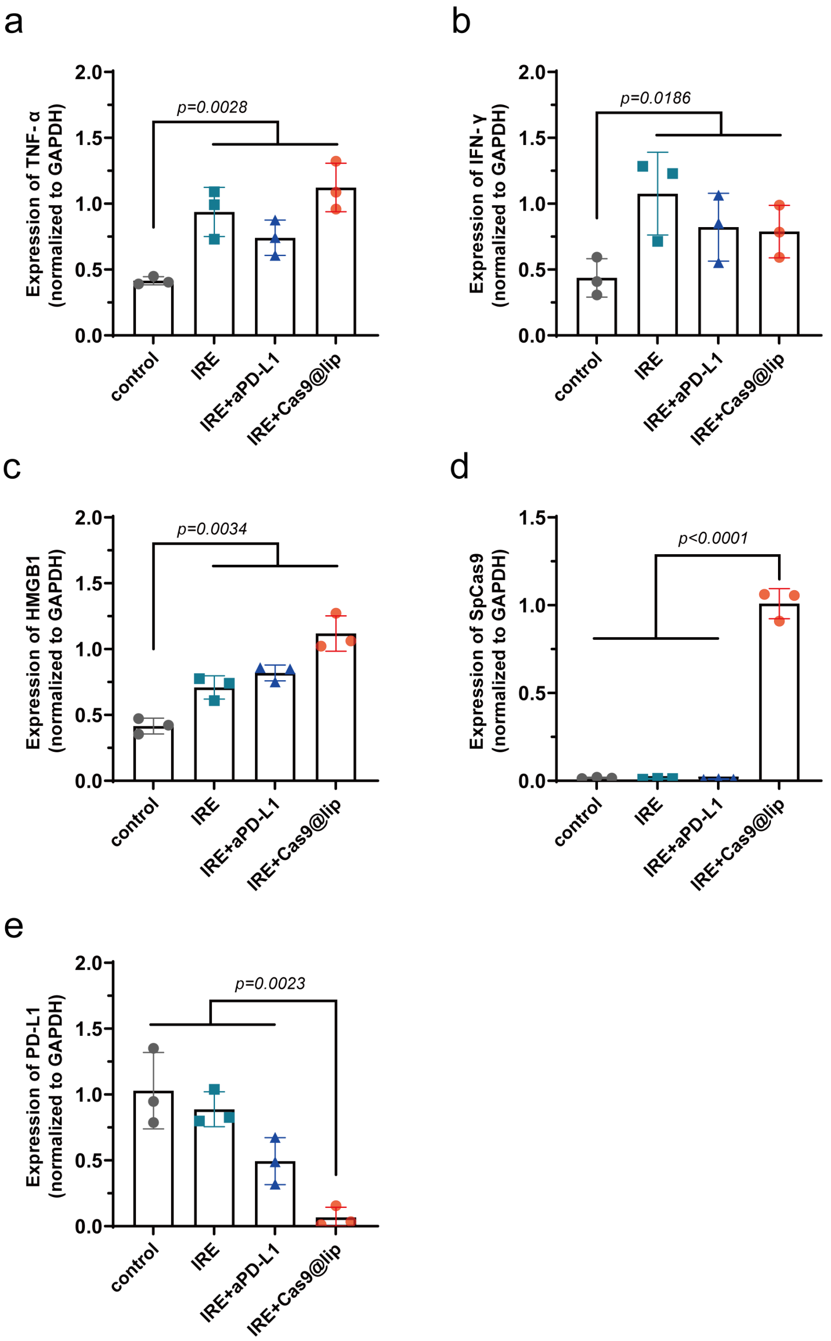

Supplementary Fig. 8. Quantification of the Western blotting. (a-e) Quantification the expression of TNF-α **(a)**, IFN-γ **(b)**, HMGB1 **(c)** and PD-L1 **(d)** and extrinsic SpCas9 **(e)** across different experimental groups. For **a-e**, n=3 biologically independent experiments. Data are presented as mean values +/- SEM. Two-tailed unpaired t-test.Source data are provided as a Source data file.

Supplementary Fig. 9

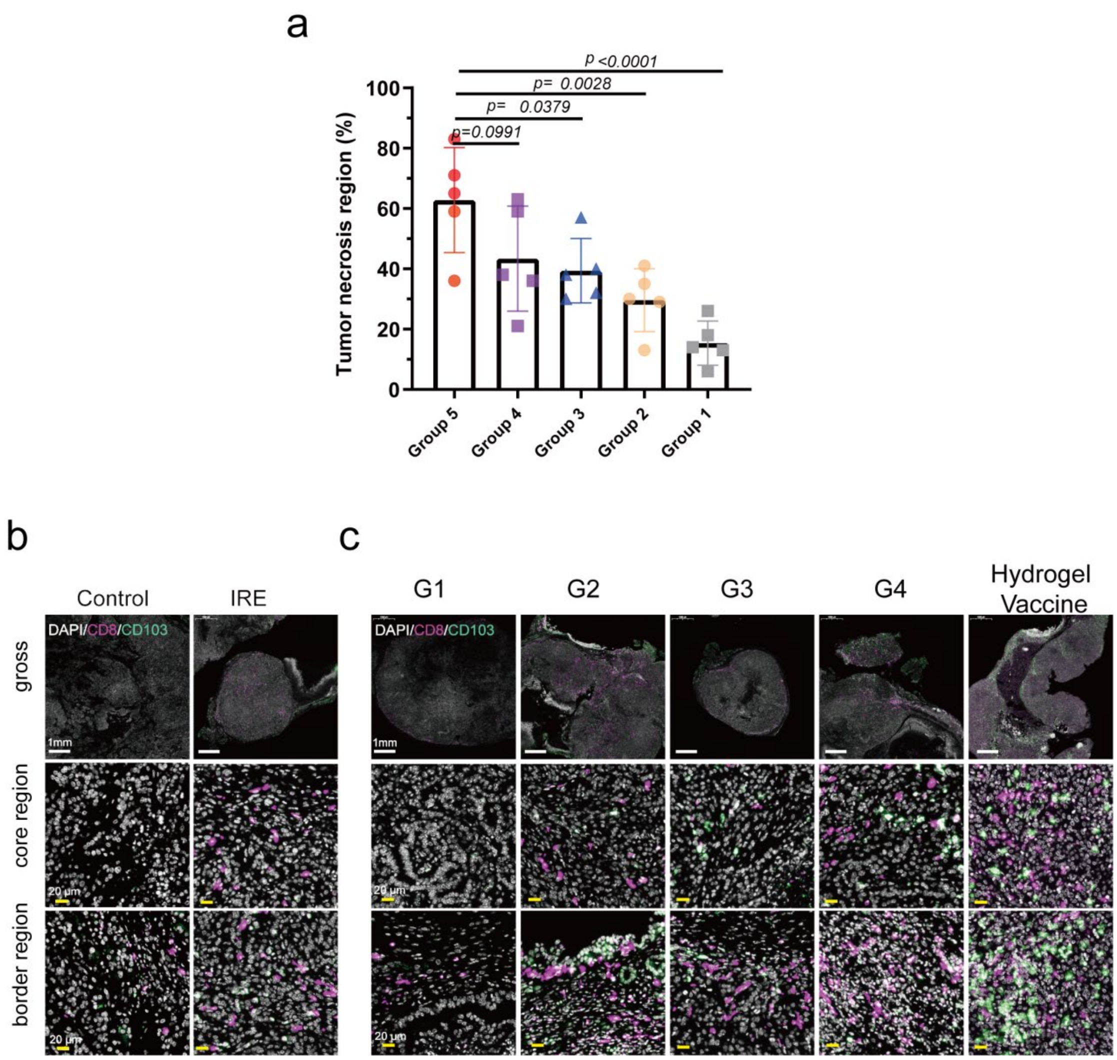

Supplementary Fig.9. Analysis the tumour necrosis, CD8^{+} and CD103^{+} immune cell infiltration across different treatment groups. (a) Statistic data showed the tumour necrosis area according to the H&E staining for tumour bearing mice treated with IRE and different hydrogel microspheres. n=5 biologically independent samples. Data are presented as mean values +/- SEM. Two-tailed Dunnett t-test. **(b-c)** Representative image of immunofluorescence staining represented the CD8^{+} and CD103^{+} immune cell infiltration across different treatment groups. For **b** and **c**, 5 biologically independent samples were performed to confirm data stability. Source data are provided as a Source data file.

Supplementary Fig. 10

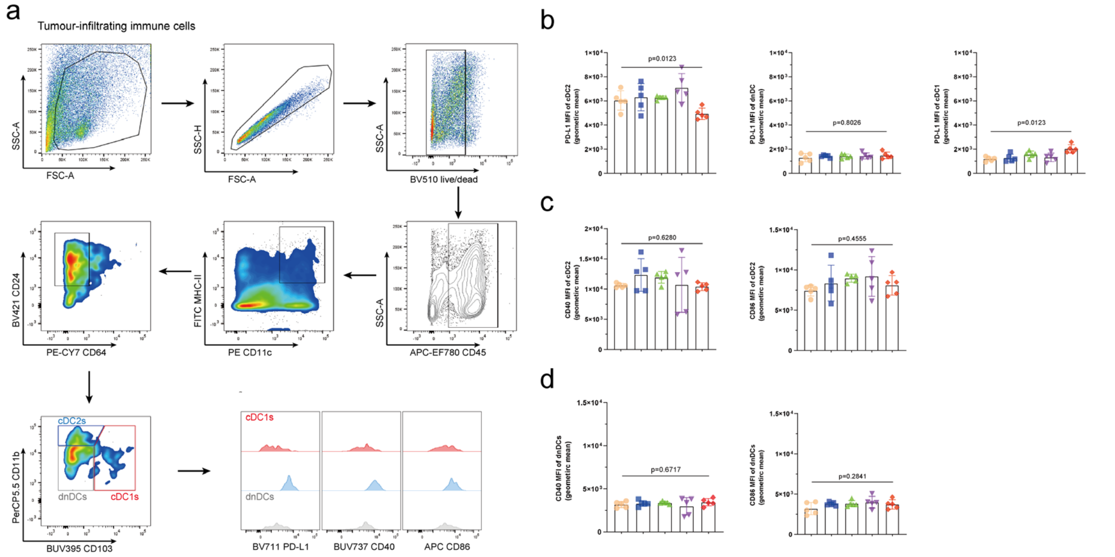

Supplementary Fig.10. Flow cytometry gating strategy for the analysis of tumour-resident DCs. (a) The gating strategy of flow cytometry experiments to identify tumour-resident $CD103^{+}CD11b^{-}$ cDC1s, $CD103^{-}CD11b^{+}$ cDC2s, $CD103^{-}CD11b^{-}$ DCs (dnDCs) and the level of costimulators (Fig 4d, e, f and Supplementary Fig.10 b-d). **(b)** Flow cytometry analysis showed that cDC2s were the major PD-L1 expressing cell. **(c)** The expression of costimulators (CD40 and CD86) on tumour-resident cDC2s across different groups. **(d)** The expression of costimulators (CD40 and CD86) on tumour-resident dnDCs across different groups. For **b-d**, n=5 biologically independent samples. Data are presented as mean values +/- SEM. Two-tailed one-way ANOVA. Source data are provided as a Source data file.

Supplementary Fig. 11

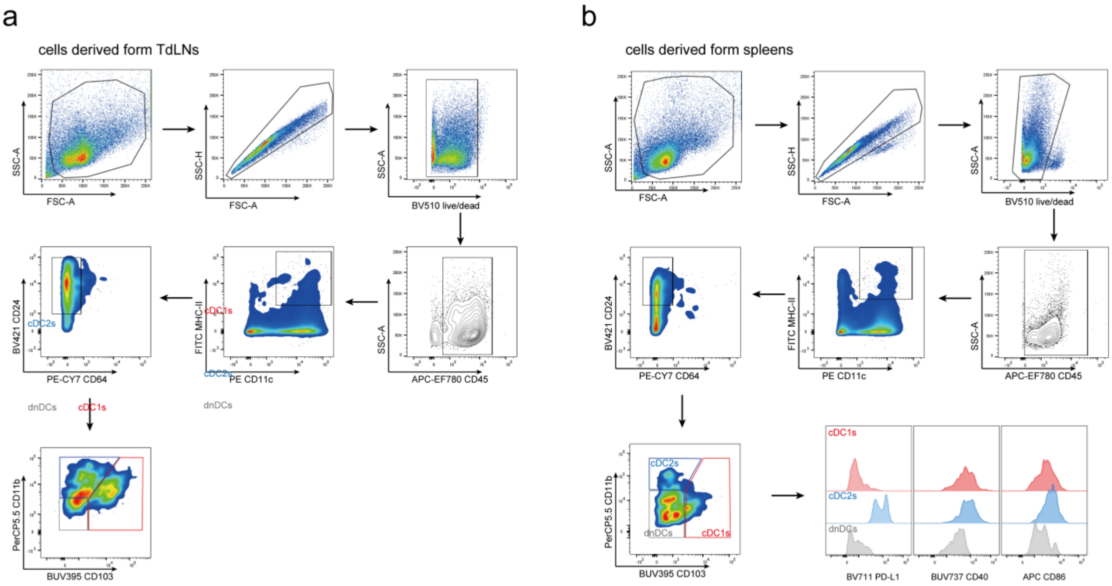

Supplementary Fig. 11. Flow cytometry gating strategy for the analysis of DCs migrated into TdLNs and spleens. (a) The gating strategy of flow cytometry experiments to identify different DCs and the level of costimulators for TdLNs (Fig. 4i, k). **(b)** The gating strategy of flow cytometry experiments to identify different DCs and the level of costimulators for spleens (Fig. 4j, l).

Supplementary Fig. 12

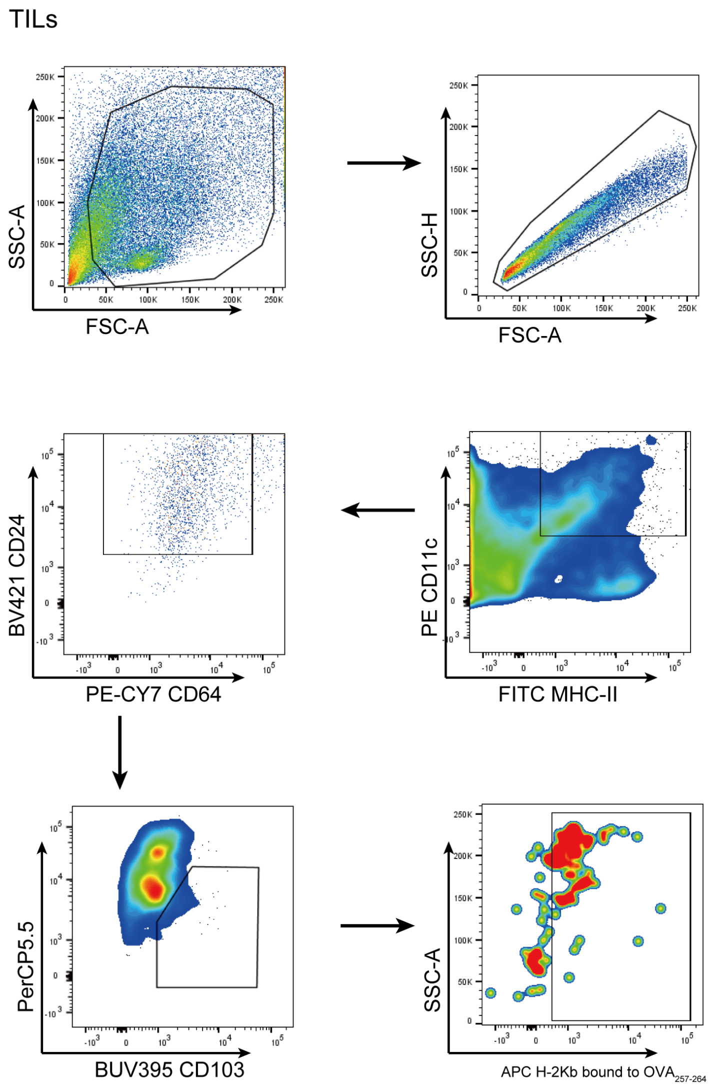

Supplementary Fig. 12. Flow cytometry gating strategy for analysis of OVA$_{257\text{-}246}$-H-2Kb specific tumour-resident cDC1s (Fig. 4n).

Supplementary Fig. 13

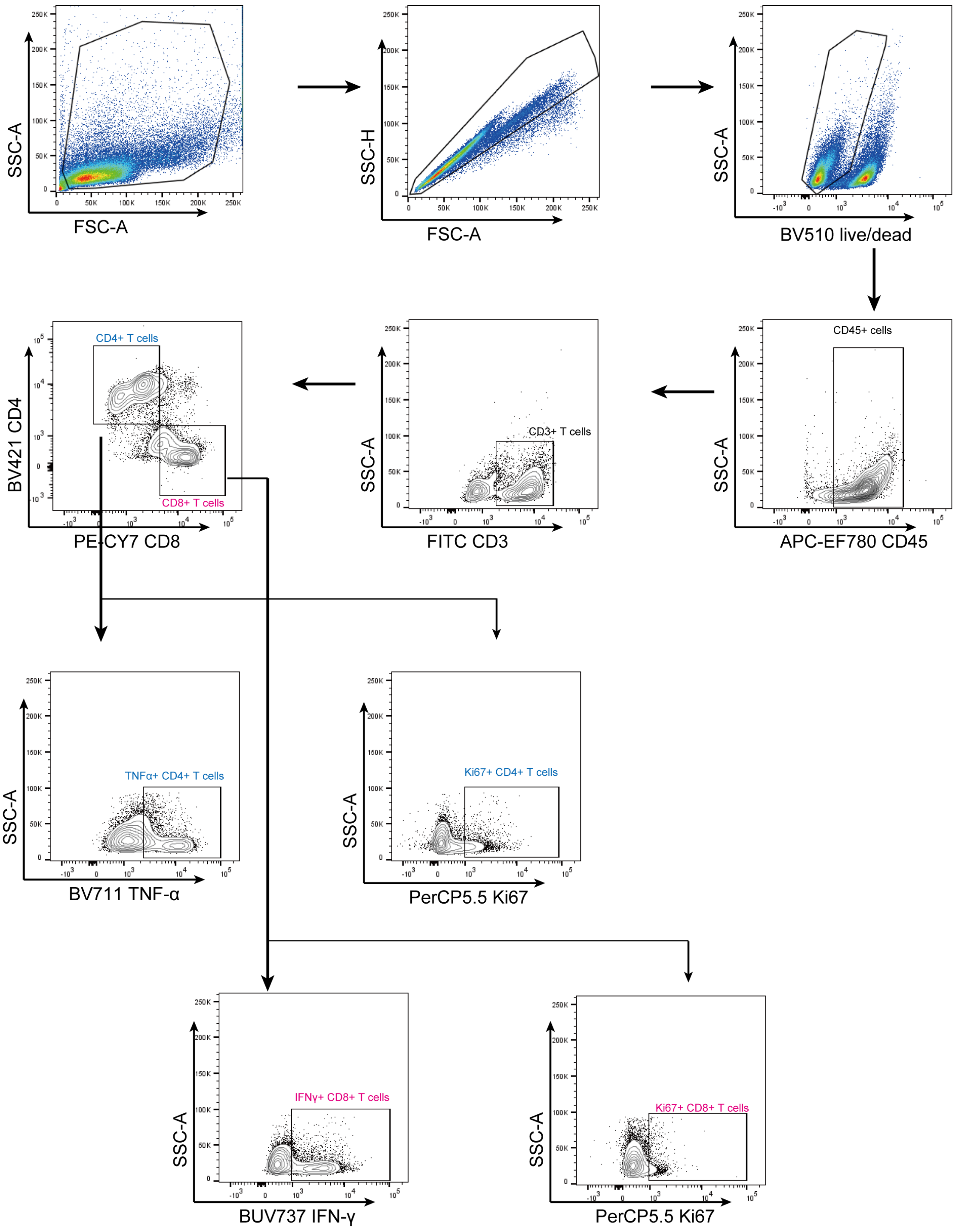

Supplementary Fig. 13. Flow cytometry gating strategy for the analysis of the intracellular expression of Ki67, IFN-γ and TNF-α in T cells (Fig. 5a, b and d-f).

Supplementary Fig. 14

a

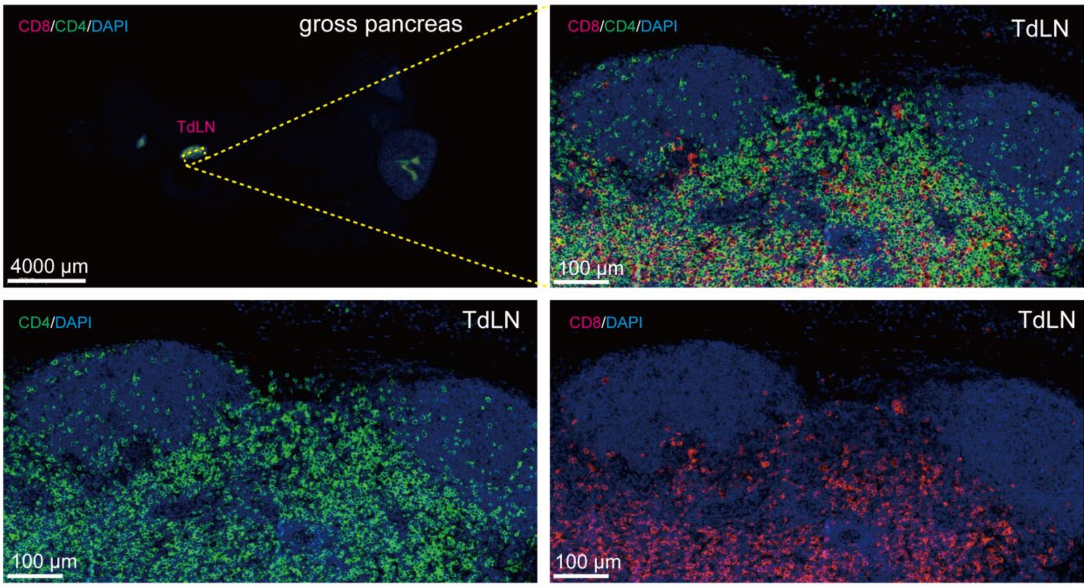

b

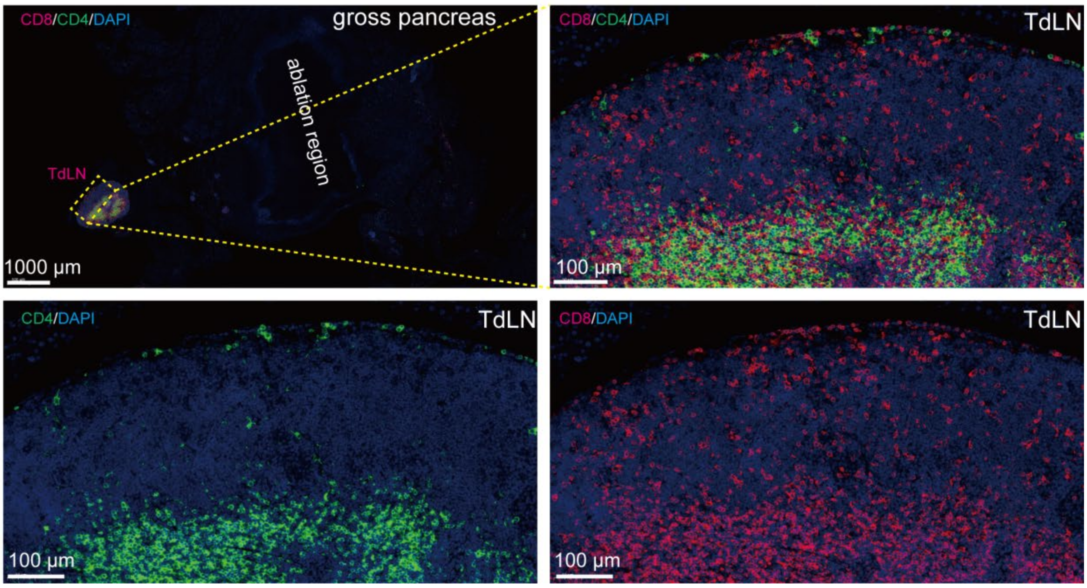

Supplementary Fig. 14. Representative images of CD8- and CD4-labelled immunofluorescence staining of pancreas lymph nodes (pLNs) in tumour bearing mice. Representative images showed CD8- and CD4-positive cells in pLNs in KPC tumour-bearing mice receiving **(a)** IRE monotherapy or **(b)** combination of IRE and hydrogel microsphere vaccine. Source data are provided as a Source data file. For **a** and **b**, 5 biologically independent samples were performed to confirm data stability.

Supplementary Fig. 15

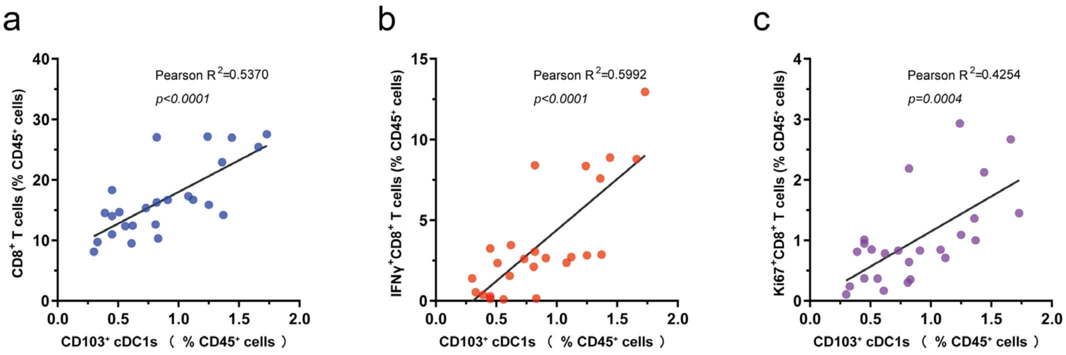

Supplementary Fig. 15. The density of cDC1s was correlated with the density of activated $CD8^+$ T cells in TdLNs. The correlation between the density of $CD8^+$ T cells (**a**), $Ki67^+CD8^+$ T cells (**b**), IFN-γ^+CD8^+ T cells (**c**) and the density of cDC1s in the TdLNs. n=25 biological independent samples, data are tested using Pearson's test with two-tailed p values. Source data are provided as a Source data file.

Supplementary Fig. 16

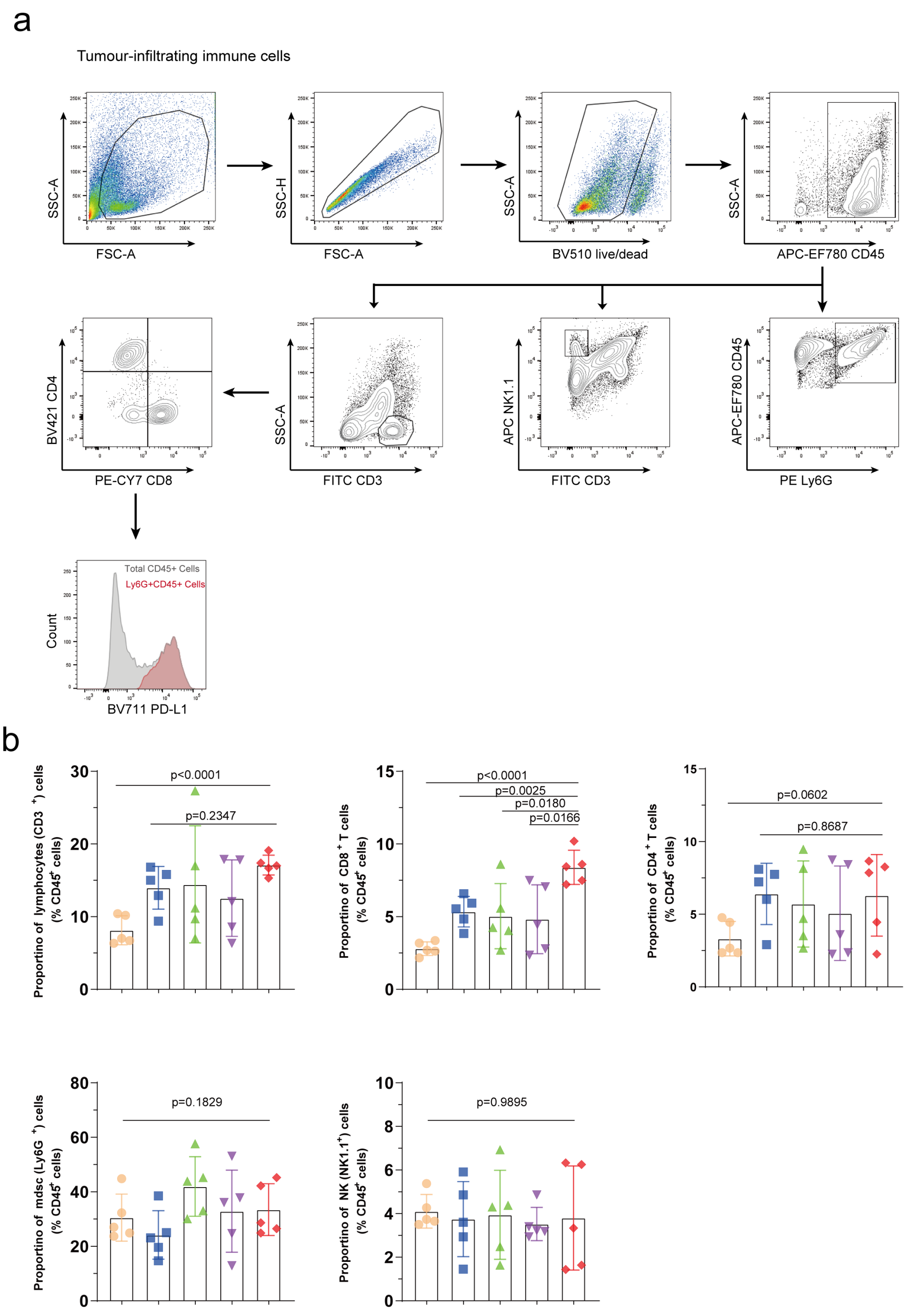

Supplementary Fig. 16. Flow cytometry gating strategy for the analysis of (a) tumour-infiltrating Ly6G^{+}CD11b^{+} myeloid cells and NK1.1^{+} cells and (b) quantification analysis (Fig. 5d-e and Supplementary Fig. 16b). For **b**, n=5 biologically independent samples. Data are presented as mean values +/- SEM. Two-tailed unpaired t-test for the upper panel, and two-tailed one-way ANOVA for the bottom panel. Source data are provided as a Source data file.

Supplementary Fig. 17

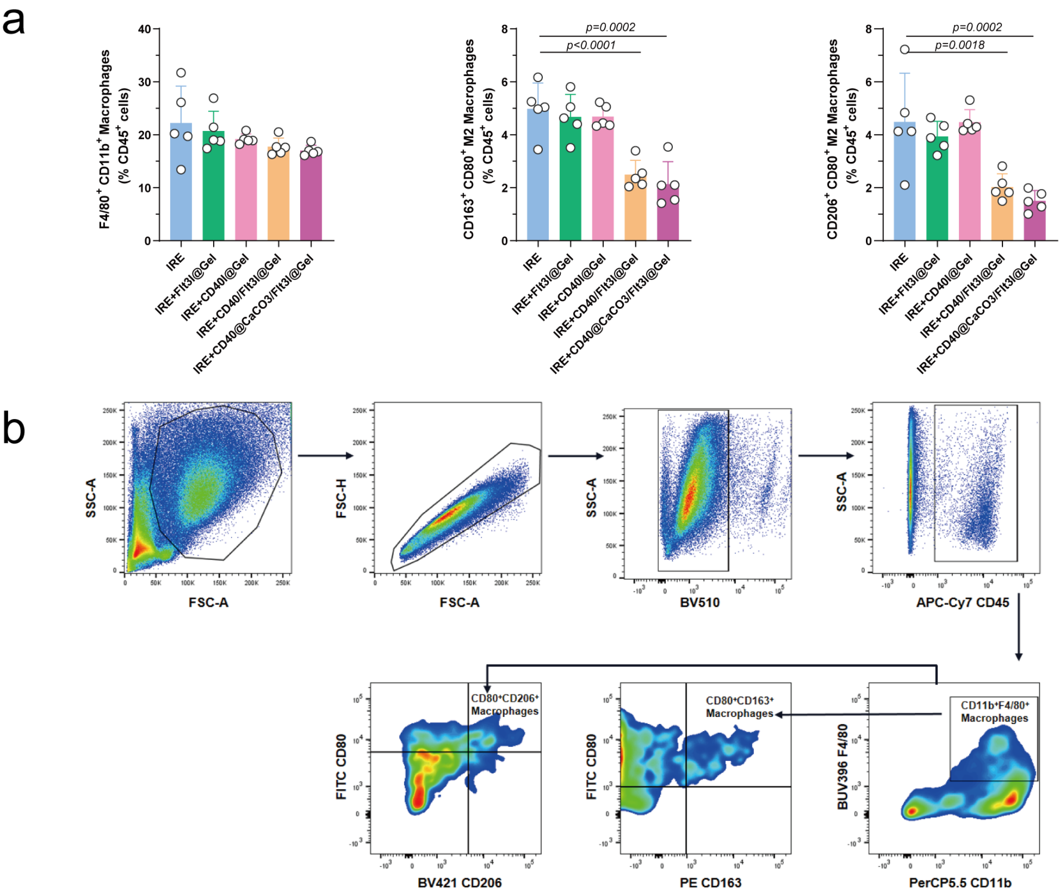

Supplementary Fig. 17. Flow cytometry gating strategy for immune phenotyping of the macrophages. (**a**) Statistical analysis revealed no differences in the proportion of total F4/80$^+$ macrophages in orthotopic pancreatic tumours across different treatment groups, while the M2-like immunosuppressive macrophages (CD163$^+$CD80$^+$ macrophages and CD206$^+$CD80$^+$ macrophages) slightly reduced after the CD40L and FLT3L combination treatment. n=5 biologically independent samples. Data are presented as mean values +/- SEM. Two-tailed Dunnett t-test. (**b**) The flow cytometry gating strategy for macrophages (Supplementary Fig. 17a). The CD206$^+$CD80$^+$ and CD163$^+$CD80$^+$ macrophage represented for the M2-like macrophages, and the CD206$^-$CD80$^+$ and CD163$^-$CD80$^+$ macrophage represented for the M2-like macrophages. Source data are provided as a Source data file.

Supplementary Fig. 18

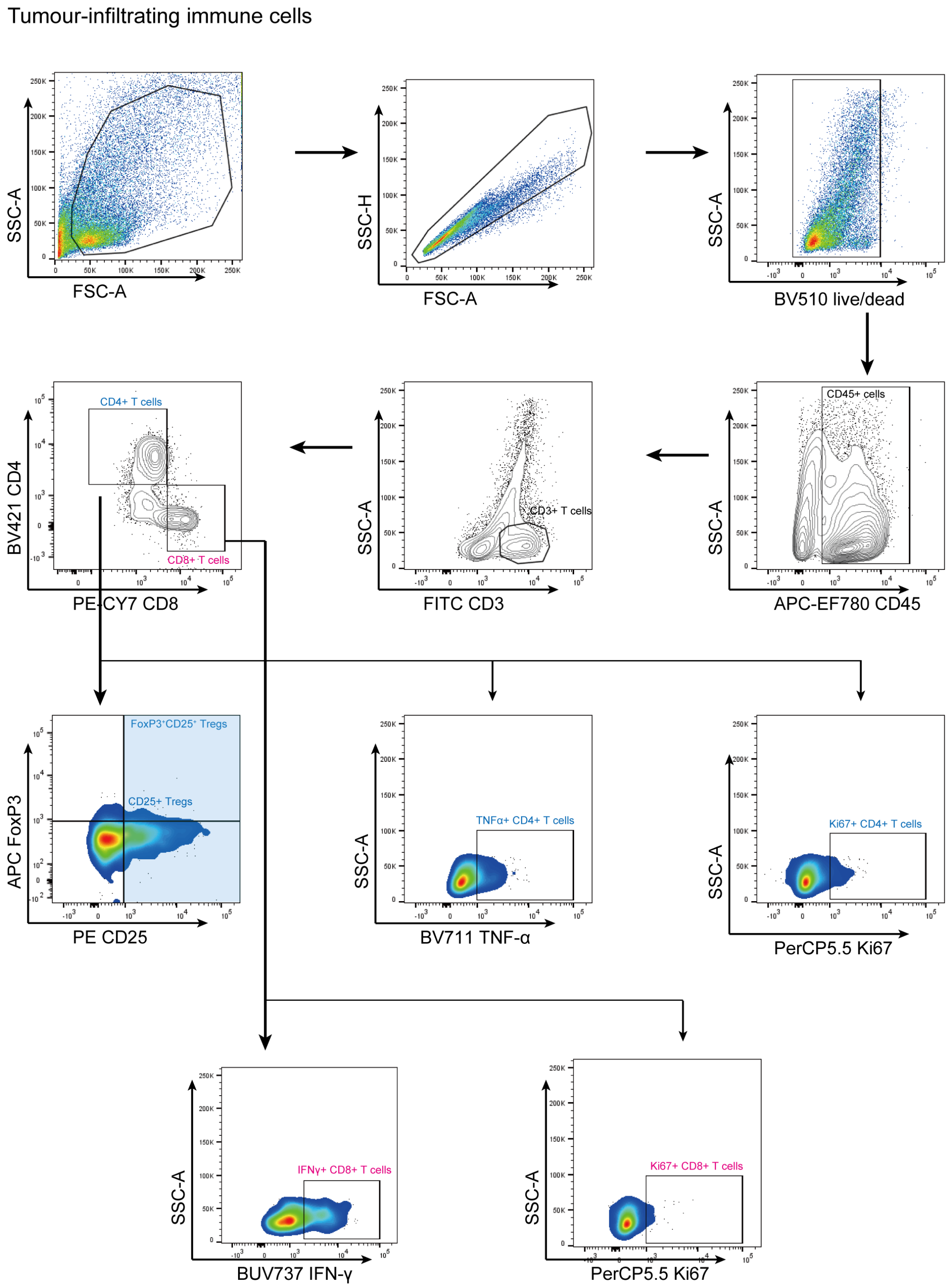

Supplementary Fig. 18. Flow cytometry gating strategy for the analysis of tumour-infiltrating FoxP3$^+$CD25$^+$ Tregs, Ki67$^+$CD8$^+$ T cells, and IFN-γ$^+$CD8$^+$ T cells (Fig. 5f) .

Supplementary Fig. 19

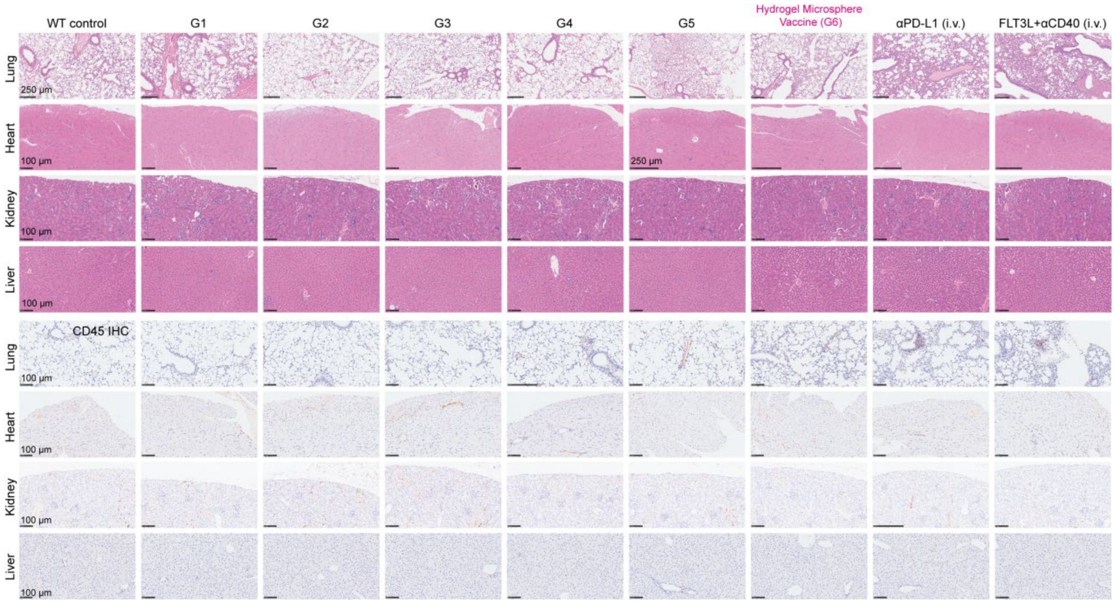

Supplementary Fig. 19. Representative H&E staining images of major organs in KPC orthotropic tumour bearing mice receiving different therapies. No obvious signs of organ damage appeared in hydrogel microspheres treated mice. Sporadic inflammatory responses were observed in the lungs of mice receiving i.p injection of αCD40 and FLT3L cytokines. 5 biologically independent samples were performed to confirm data stability. Source data are provided as a Source data file.

Supplementary Fig. 20

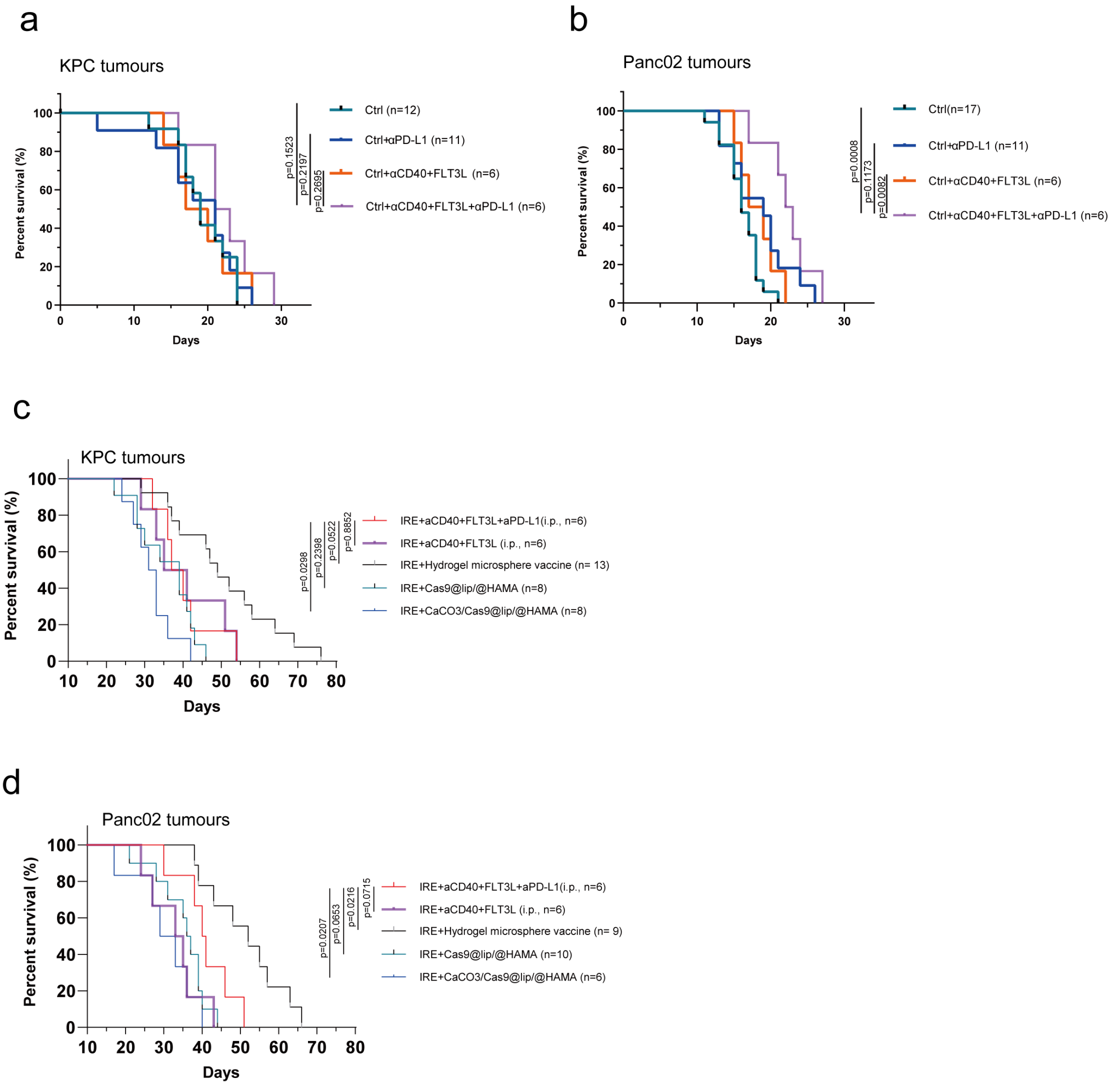

Supplementary Fig. 20. The efficacy of intravenous delivery of αCD40 and FLT3L against KPC tumour. The survival data of control group, control+αPD-L1 group **(a-b)**, IRE+vaccine (different counterparts in **c-d**) group were related to survival analyses in **Fig. 3** and **Fig. 6**. Sample size for each group is labelled in the figure. Survival data are analyzed using Kaplan-Meier Method. Two-tailed log-rank test. Source data are provided as a Source data file.

Supplementary Fig. 21

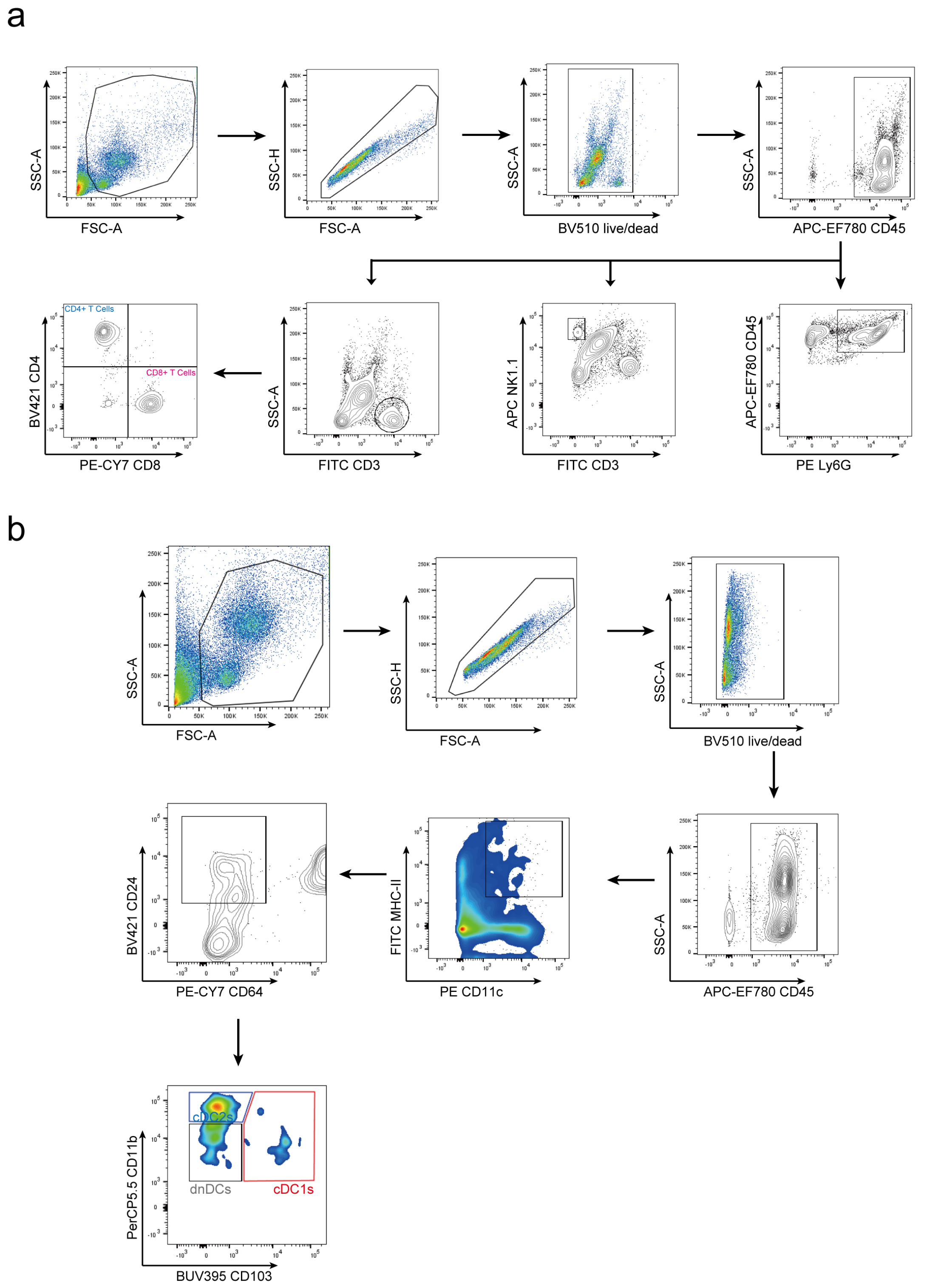

Supplementary Fig. 21. Flow cytometry gating strategy for the analysis of CD8^{+} T cells, CD4^{+} T cells (a), CD24^{+}MHC II^{+}DC-like cells and CD103^{+} cDC1-like cells (b) in circulation (Fig. 6g).

Supplementary Fig. 22

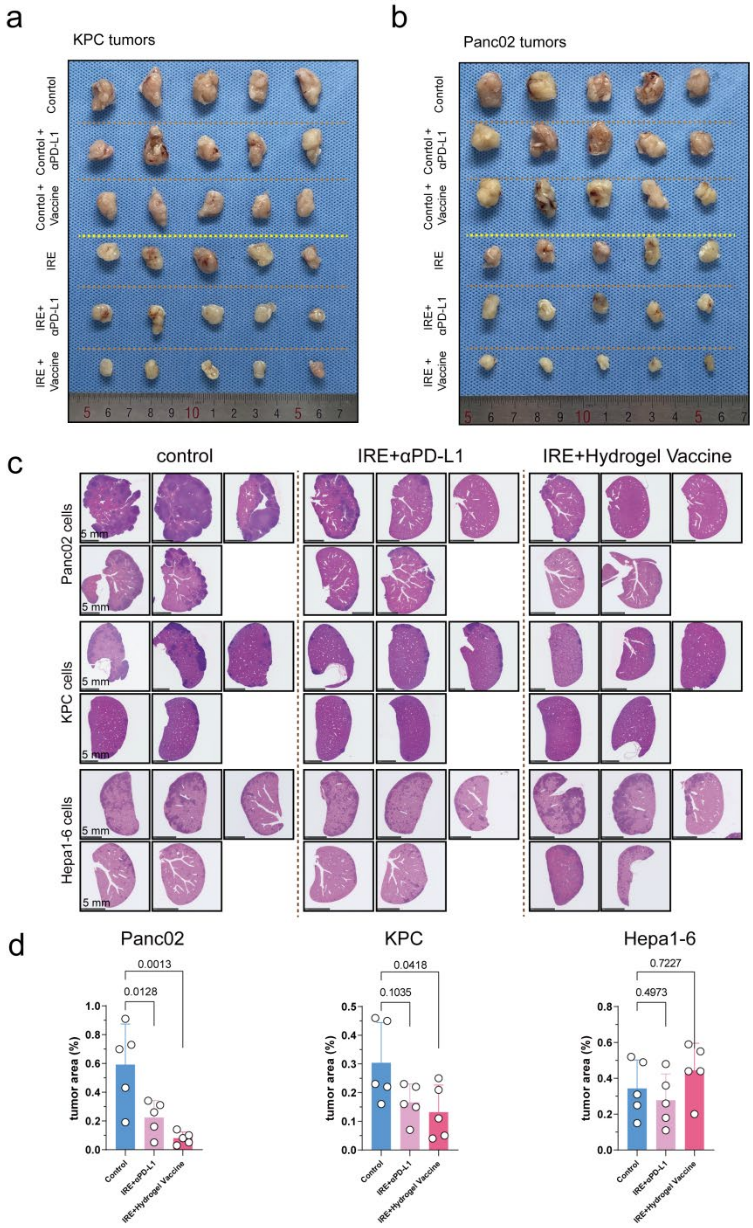

Supplementary Fig. 22. Representative images show the gross distant metastasis tumours across different groups. (**a, b**) Representative images show the subcutaneous metastasis KPC (**a**) and Panc02 (**b**) tumours across different treatment groups. (**c**) Representative images show the Panc02, KPC, and Hepa1-6 liver metastasis tumours across different groups. (**d**) Statistical analysis showed that the hydrogel microsphere vaccine combined with IRE ablation significantly the tumour area in KPC and Panc02 induced liver metastases. n=5 biologically independent samples. Data are presented as mean values +/- SEM. Two-tailed Dunnett t-test. Source data are provided as a Source data file.

Supplementary Fig. 23

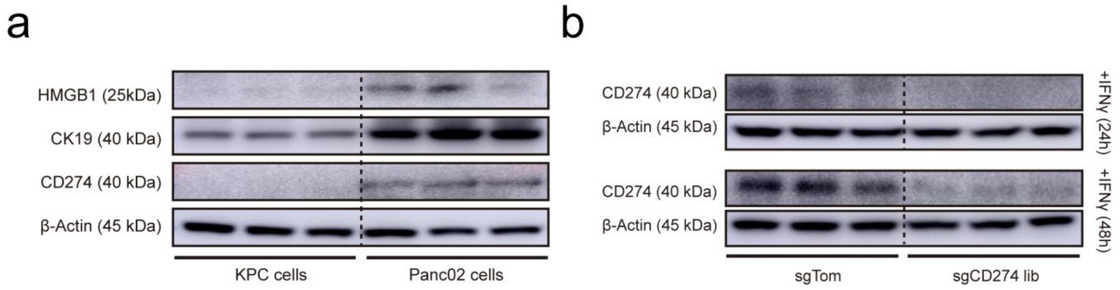

Supplementary Fig. 23. Western Blot assay demonstrating the expression level molecular markers in KPC and Panc02 cells, and the expression of PD-L1 in IFN-γ treatment Panc02 cells.

(**a**) Western Blot assay demonstrating the expression level of HMGB1, CK19, and CD274 (PD-L1) in KPC and Panc02 cells. (**b**) The expression of PD-L1 in IFN-γ treatment Panc02 cells. For a and b, 3 biologically independent samples were performed to confirm data stability. Source data are provided as a Source data file.

Supplementary Table 1. Antibody information.

Antibodies	Source	Reactivity	Provider	Catalog	Application
PD-L1	Rat	Mouse	BioXCell	BP0101	in vivo treatment
IgG1 isotype	Rat	Mouse	BioXCell	BP0090	in vivo treatment
CD40	Rat	Mouse	BioXCell	BP0016-2	in vivo treatment
FLT3L	Mouse	Mouse	Sino Biological	51113-M02H	in vivo treatment
CD8β	Rat	Mouse	BioXCell	BE0223	in vivo treatment
MHC I	Mouse	Mouse	BioXCell	BE0172	in vivo treatment
CD45	Mouse	Human, Pig, Mouse, Rat	ProteinTech	60287-1-Ig	IHC (1:4000), IF (1:50)
FoxP3	Rabbit	Mouse, Monkey	Cell Signalling Technology	12653	IHC ((1:200), IF (1:400)
CD8	Rabbit	Mouse	Abcam	ab209775	IHC (1:2000)
CD4	Rabbit	Mouse	Abcam	ab183685	IHC (1:1000)
CD103	Rabbit	Mouse, Rat, Human	Abcam	ab224202	IHC (1:1000) IF (1:50)
CK19	Rabbit	Human, Mouse, Rat, Dog, Goat, Pig	ProteinTech	10712-1-AP	IHC (1:3000), IF (1:200)
CD31	Rabbit	Mouse, Rat, Human	Abcam	ab182981	IHC (1:2000) IF (1:100)
CK19	Mouse	Human, Mouse, Rat, Pig	ProteinTech	60187-1-Ig	WB (1:20000)

TNF-α	Mouse	Human, Mouse, Rat, Bovine, Chicken, Monkey, Pig	ProteinTech	60291-1-Ig	WB (1:1000)
IFN-γ	Goat	Mouse, Rat	Invitrogen	PA1-24782	WB (1:1000)
HMGB1	Rabbit	Mouse, Rat, Human	Abcam	ab18256	WB (1:1000)
SpCas9	Rabbit	Recombinant fragment	Abcam	ab189380	WB (1:20000)
β-Actin	Mouse	Human, Mouse, Rat, Hamster, Dog	Cell Signalling Technology	3700	WB (1:1000)
GAPDH	Mouse	Mouse, Rat, Chicken, Cow, Human, Pig, Xenopus laevis, Chinese hamster	Abcam	ab9484	WB (1:10000)
PD-L1	Mouse	Human, Mouse, Pig, Rat	ProteinTech	66248-1-Ig	WB (1:2000)
APC-eFluor™780 CD45	Rat	Human, Mouse	eBioscience	47-0451-82	FC (1:50)
FITC CD3	Rat	Bacteria, Fungi, Mouse, Rat	eBioscience	11-0032-82	FC (1:50)
PE-Cyanine7 CD8A	Rat	Human, Mouse	eBioscience	25-0081-81	FC (1:50)
BV421 CD4	Rat	Mouse	BD Biosciences	562891	FC (1:50)
PE-eFluor™ 610 CD11B	Rat	Fish, Human, Mouse	eBioscience	61-0112-80	FC (1:50)
PE Ly-6G	Rat	Mouse	BD Biosciences	551461	FC (1:50)
APC NK-1.1	Mouse	Mouse	BD Biosciences	550627	FC (1:50)

BUV395 CD273	Rat	Mouse	BD Biosciences	565102	FC (1:50)
Brilliant Violet 711™ CD274	Rat	Mouse	Biolegend	124319	FC (1:50)
PE CD25	Rat	Mouse, Rat	eBioscience	12-0251-82	FC (1:50)
PE Granzyme B	Mouse	Human, Mouse	Biolegend	372207	FC (1:50)
APC FOXP3	Rat	Dog, Horse, Human, Mouse, Pig, Rat	eBioscience	17-5773-80	FC (1:50)
V450 Ki-67	Mouse	Human, Mouse	BD Biosciences	561281	FC (1:50)
Brilliant Violet 711™ TNF-α	Rat	Mouse	Biolegend	506349	FC (1:50)
BUV737 IFN-γ	Rat	Mouse	BD Biosciences	612769	FC (1:50)
PE CD152	Armenian Hamster	Mouse	Biolegend	106305	FC (1:50)
PerCP/Cyanine5.5 CD223 (LAG-3)	Rat	Mouse	Biolegend	125212	FC (1:50)
Brilliant Violet 711™ CD366	Rat	Mouse	Biolegend	119727	FC (1:50)
FITC I-A/I-E	Rat	Mouse	Biolegend	107606	FC (1:50)
PE/Cyanine7 CD64	Mouse	Mouse	Biolegend	139314	FC (1:50)
Brilliant Violet 421™ CD24	Rat	Mouse	Biolegend	101826	FC (1:50)
PE CD11c	Armenian Hamster	Mouse	Biolegend	117308	FC (1:50)
APC H-2Kb bound to SIINFEKL	Mouse	Mouse	Biolegend	141605	FC (1:50)
BUV395 CD103	Rat	Mouse	BD Biosciences	740238	FC (1:50)
APC H-2Kd	Mouse	Mouse	Biolegend	116620	FC (1:50)

APC H-2Kb	Mouse	Mouse	Biolegend	116518	FC (1:50)
APC CD86	Rat	Mouse	Biolegend	105012	FC (1:50)
Brilliant Violet 711™ CD80	Armenian Hamster	Mouse, Dog	Biolegend	104743	FC (1:50)
BUV737 CD40	Rat	Mouse	BD Biosciences	741749	FC (1:50)
FITC CD80	Armenian Hamster	Mouse, Dog	BioLegend	104706	FC (1:50)
PE/Cyanine7 CD86	Rat	Mouse	BioLegend	105014	FC (1:50)
Brilliant Violet 421™ CD206	Rat	Mouse	BioLegend	141717	FC (1:50)
PE CD163	Rat	Mouse	BioLegend	155308	FC (1:50)
PerCP-Cy™5.5 CD11b	Rat	Human, Mouse	eBioscience	61-0112-80	FC (1:50)
BUV395 F4/80	Rat	Mouse	BD Biosciences	565614	FC (1:50)
