# Supplementary material for: Interventional hydrogel microsphere vaccine as an immune amplifier for activated antitumour immunity after ablation therapy
Source: Nat Commun. 2023 Jul 11;14:4106. doi: 10.1038/s41467-023-39759-w (PMC10336067; doi:10.1038/s41467-023-39759-w)
Supplement: Supplementary file 3 — Reporting Summary [file 41467_2023_39759_MOESM3_ESM.pdf]

nature portfolio

Corresponding author(s): Wenguo Cui, Zhongmin Wang

Last updated by author(s): May 28, 2023

Reporting Summary

Nature Portfolio wishes to improve the reproducibility of the work that we publish. This form provides structure for consistency and transparency in reporting. For further information on Nature Portfolio policies, see our Editorial Policies and the Editorial Policy Checklist.

Statistics

For all statistical analyses, confirm that the following items are present in the figure legend, table legend, main text, or Methods section.

n/a	Confirmed	
☐	☒	The exact sample size (*n*) for each experimental group/condition, given as a discrete number and unit of measurement
☐	☒	A statement on whether measurements were taken from distinct samples or whether the same sample was measured repeatedly
☐	☒	The statistical test(s) used AND whether they are one- or two-sided<br>*Only common tests should be described solely by name; describe more complex techniques in the Methods section.*
☒	☐	A description of all covariates tested
☐	☒	A description of any assumptions or corrections, such as tests of normality and adjustment for multiple comparisons
☐	☒	A full description of the statistical parameters including central tendency (e.g. means) or other basic estimates (e.g. regression coefficient) AND variation (e.g. standard deviation) or associated estimates of uncertainty (e.g. confidence intervals)
☐	☒	For null hypothesis testing, the test statistic (e.g. *F*, *t*, *r*) with confidence intervals, effect sizes, degrees of freedom and *P* value noted<br>*Give P values as exact values whenever suitable.*
☒	☐	For Bayesian analysis, information on the choice of priors and Markov chain Monte Carlo settings
☒	☐	For hierarchical and complex designs, identification of the appropriate level for tests and full reporting of outcomes
☐	☒	Estimates of effect sizes (e.g. Cohen's *d*, Pearson's *r*), indicating how they were calculated

Our web collection on statistics for biologists contains articles on many of the points above.

Software and code

Policy information about availability of computer code

Data collection	he initial sample size was estimated using the PASS software (Version 2015). The transcriptome profiles (Htseq-counts) and clinical data of the TCGA cohort were obtained using the R TCGAbiolinks package (version 2.20.1). The NanoZoomer S360 (Hamamatsu Photonics, Hamamatsu, Japan) was used to scan slides of IHC-staining, and the Pannoramic MIDI was used to scan slides of IF-staining (3DHISTECH Digital Pathology Company, Budapest, Hungary). The flow-cytometry experiments were performed using BD LSR Fortessa X-20 instrument. The IVIS spectrum imager (IVIS Lumina III, PerkinElmer, CLS136334) was used for collection of bioluminescence data, and Living Image Software (Living Imaging, PerkinElmer, version 4.2) was used for collection of radiance intensity.
Data analysis	Statistical analysis was performed using R (version 3.6.3) and GraphPad Prism 8 software (San Diego, CA, USA). The fastp (version 0.20.1), Bowtie2 (version 2.2.4) software and DEseq2 package (version 3.12, R version 3.6.3) were used to identify the differentially expressed genes between IRE treated or non-IRE treated tumors. The R BioMart package (version 0.7) was used to convert the murine gene IDs to the corresponding human gene IDs, and then GSEApy software (version 0.9.18) was used for pre-ranked GSEA analysis. The R GSVA package (version 1.40.1) was used for the single-sample gene set enrichment analysis to identify the infiltration feature of immune cells. The R ConsensusClusterPlus Package (version 1.58.0) and factoextra package (version 1.0.7) were used to identify the unsupervised clustering of the immune-infiltrating data. The flow cytometry data were analyzed using FlowJo software (version 10.7.1). The reads-count to TPM convert script are fully publicly available (https://github.com/sdtaliuxiaoyu/read-counts-to-TPM.git).

For manuscripts utilizing custom algorithms or software that are central to the research but not yet described in published literature, software must be made available to editors and reviewers. We strongly encourage code deposition in a community repository (e.g. GitHub). See the Nature Portfolio guidelines for submitting code & software for further information.

Data

Policy information about availability of data

All manuscripts must include a data availability statement. This statement should provide the following information, where applicable:

- Accession codes, unique identifiers, or web links for publicly available datasets
- A description of any restrictions on data availability
- For clinical datasets or third party data, please ensure that the statement adheres to our policy

The raw sequencing data generated in this study have been deposited in the NCBI Gene Expression Omnibus (GEO) database under accession code GSE215417 [https://www.ncbi.nlm.nih.gov/geo/query/acc.cgi?acc=GSE215417]. The data and detailed information of the experiments are fully publicly available. The remaining data generated in this study are provided in the Supplementary Information and Source Data file. Source data are provided with this paper.

Research involving human participants, their data, or biological material

Policy information about studies with human participants or human data. See also policy information about sex, gender (identity/presentation), and sexual orientation and race, ethnicity and racism.

Reporting on sex and gender	Not applicable.
Reporting on race, ethnicity, or other socially relevant groupings	Not applicable.
Population characteristics	Not applicable.
Recruitment	Not applicable.
Ethics oversight	Not applicable.

Note that full information on the approval of the study protocol must also be provided in the manuscript.

Field-specific reporting

Please select the one below that is the best fit for your research. If you are not sure, read the appropriate sections before making your selection.

☒ Life sciences ☐ Behavioural & social sciences ☐ Ecological, evolutionary & environmental sciences

For a reference copy of the document with all sections, see nature.com/documents/nr-reporting-summary-flat.pdf

Life sciences study design

All studies must disclose on these points even when the disclosure is negative.

Sample size	The initial sample size was estimated using the PASS software (Version 2015). According to the main purpose of the study to estimate the proportion change of CD103+ cDC1s between the treatment group and the control group, two-sample T-tests (Assuming Equal Variance) were used. The parameters were set as: test direction = two-sided; test power = 0.85, input type = means, mean1=50, mean2=35 (CD103+ cDC1s / total DCs proportion), standard deviation=8. The results showed that the minimum sample size for each group was 5. The sample size of the entire study was based on the calculations with necessary modifications, and the sample sizes were increased in survival analysis.
Data exclusions	Mice that died within 5 days of IRE surgery were considered as surgical failures, and were not included in the follow-up study for pancreatic cancer immunotherapies.
Replication	For in vitro experiments, each experimental group was repeated at least 3 times for statistical analysis, and all attempts at replication were successful. For in vivo experiments, 5 samples were included in each group as replicates. Only mice that survived the ablation surgery were included in the ablation group, and all attempts for downstream analysis at replication were successful.
Randomization	Mice bearing orthotopic pancreatic tumors (KPC-OVA, Panc02-OVA, KPC-LUC, or Panc02-LUC) were randomly assigned to different treatment groups according to random numbers, to receive Hydrogel Microsphere Vaccine, G1, G2, G3, or G4 injections after IRE ablation.
Blinding	Blinding is not necessary in the animal experiments according to the study design. The blinding is also impossible because the experimental group will receive surgical resection and interventional ablation therapies .

Reporting for specific materials, systems and methods

We require information from authors about some types of materials, experimental systems and methods used in many studies. Here, indicate whether each material, system or method listed is relevant to your study. If you are not sure if a list item applies to your research, read the appropriate section before selecting a response.

Materials & experimental systems

n/a	Involved in the study
☐	☒ Antibodies
☐	☒ Eukaryotic cell lines
☒	☐ Palaeontology and archaeology
☐	☒ Animals and other organisms
☒	☐ Clinical data
☒	☐ Dual use research of concern
☒	☐ Plants

Methods

n/a	Involved in the study
☒	☐ ChIP-seq
☐	☒ Flow cytometry
☒	☐ MRI-based neuroimaging

Antibodies

Antibodies used

The following staining protocols were designed to present: (1) overall distribution of immune cell, (2) T cell function, (3) identification of DC cell subsets, (4) identification of DC cell function, (5) identification the M1/M2 phenotype of tumour-infiltrating macrophages and identification of co-stimulatory molecules, respectively. Panel-1: Fixable Viability Stain 510 (1:1000 dilution, BD Biosciences, Cat. #564406, RRID: AB_2869572), APC-eFluor™780 Rat anti-mouse CD45 Antibody (1:50 dilution, eBioscience, Cat. #47-0451-82, Clone: 30-F11, RRID: AB_1548781), FITC Rat anti-mouse CD3 Antibody (1:50 dilution, eBioscience, Cat. #11-0032-82, Clone: 17A2, RRID: AB_2572431), PE-Cyanine7 Rat anti-mouse CD8A Antibody (1:50 dilution, eBioscience, Cat. #25-0081-81, Clone: 53-6.7, RRID: AB_469583), BV421 Rat anti-Mouse CD4 Antibody (1：50 dilution, BD Biosciences, Cat. #562891, Clone: GK1.5, RRID:AB_2737870), PE-eFluor™ 610 Rat anti-mouse CD11B Antibody (1:50 dilution, eBioscience, Cat. #61-0112-80, Clone: M1/70, RRID: AB_2574527), PE Rat anti-mouse Ly-6G Antibody (1:50 dilution, BD Biosciences, Cat. #551461, Clone: 1A8, RRID: AB_394208), APC Mouse anti-mouse NK-1.1 Antibody (1:50 dilution, BD Biosciences, Cat. #550627, Clone: PK136, RRID: AB_398463), BUV395 Rat anti-mouse CD273 Antibody (1：50 dilution, BD Biosciences, Cat. #565102, Clone: TY25, RRID: AB_2739068), Brilliant Violet 711™ Rat anti-mouse CD274 (B7-H1, PD-L1) Antibody (1:50 dilution, BioLegend, Cat. #124319, Clone: 10F.9G2, RRID: AB_2563619). Panel-2: Fixable Viability Stain 510 (1:1000 dilution, BD Biosciences, Cat. #564406, RRID: AB_2869572), APC-eFluor™780 Rat anti-mouse CD45 Antibody (1:50 dilution, eBioscience, Cat. #47-0451-82, Clone: 30-F11, RRID: AB_1548781), FITC Rat anti-mouse CD3 Antibody (1:50 dilution, eBioscience, Cat. #11-0032-82, Clone: 17A2, RRID: AB_2572431), PE-Cyanine7 Rat anti-mouse CD8A Antibody (1:50 dilution, eBioscience, Cat. #25-0081-81, Clone: 53-6.7, RRID: AB_469583), BV421 Rat anti-Mouse CD4 Antibody (1:50 dilution, BD Biosciences, Cat. #562891, Clone: GK1.5, RRID: AB_2737870), PE Rat anti-mouse CD25 Antibody (1:100 dilution, eBioscience, Cat. #12-0251-82, Clone: PC61.5, RRID: AB_465607) or PE Mouse anti-human/mouse Granzyme B Recombinant Antibody (1:50 dilution, BioLegend, Cat. #372207, Clone: QA16A02, RRID: AB_2687031), APC Rat anti-human/mouse FOXP3 Antibody (1:50 dilution, eBioscience, Cat. #17-5773-80, Clone: FJK-16s, RRID: AB_469456), V450 Mouse anti-mouse/human Ki-67 Antibody (1:50 dilution, BD Biosciences, Cat. #561281, Clone: B56, RRID: AB_10613816), Brilliant Violet 711™ Rat anti-mouse TNF-α Antibody (1:50 dilution, BioLegend, Cat. #506349, Clone: MP6-XT22, RRID: AB_2629800), BUV737 Rat Anti-Mouse IFN-γ Antibody (1:50 dilution, BD Biosciences, Cat. #612769, Clone: XMG1.2). Panel-3: Fixable Viability Stain 510 (1:1000 dilution, BD Biosciences, Cat. #564406, RRID: AB_2869572), APC-eFluor™780 Rat anti-mouse CD45 Antibody (1:50 dilution, eBioscience, Cat. #47-0451-82, Clone: 30-F11, RRID: AB_1548781), FITC Rat anti-mouse CD3 Antibody (1:50 dilution, eBioscience, Cat. #11-0032-82, Clone: 17A2, RRID: AB_2572431), PE-Cyanine7 Rat anti-mouse CD8A Antibody (1:50 dilution, eBioscience, Cat. #25-0081-81, Clone: 53-6.7, RRID: AB_469583), BV421 Rat anti-Mouse CD4 Antibody (1:50 dilution, BD Biosciences, Cat. #562891, Clone: GK1.5, RRID: AB_2737870), PE Armenian Hamster anti-mouse CD152 Antibody (1:50 dilution, BioLegend, Cat. #106305, Clone: UC10-4B9, RRID: AB_313254), APC Mouse anti-mouse NK-1.1 Antibody (1:50 dilution, BD Biosciences, Cat. #550627, Clone:PK136, RRID: AB_398463), PerCP/Cyanine5.5 Rat anti-mouse CD223 (LAG-3) Antibody (1:50 dilution, BioLegend, Cat. #125212, Clone: C9B7W, RRID: AB_2561517), Brilliant Violet 711™ Rat anti-mouse CD366 (Tim-3) Antibody (1:50 dilution, BioLegend, Cat. #119727, Clone: RMT3-23, RRID: AB_2716208), BUV395 Rat anti-Mouse CD273 Antibody (1:50 dilution, BD Biosciences, Cat. #565102, Clone: TY25, RRID: AB_2739068). Panel-4.1: Fixable Viability Stain 510 (1:1000 dilution, BD Biosciences, Cat. #564406, RRID: AB_2869572), APC-eFluor™780 Rat anti-mouse CD45 Antibody (1:50 dilution, eBioscience, Cat. #47-0451-82, Clone: 30-F11, RRID: AB_1548781), FITC Rat anti-mouse I-A/I-E Antibody (1:50 dilution，BioLegend, Cat. #107606, Clone: M5/114.15.2, RRID: AB_313321), PE/Cyanine7 Mouse anti-mouse CD64 (FcγRI) Antibody (1:50 dilution, BioLegend, Cat. #139314, Clone: X54-5/7.1, RRID:AB_2563904), Brilliant Violet 421™ Rat Anti-mouse CD24 Antibody (1:50 dilution, BioLegend, Cat. #101826, Clone: M1/69, RRID: AB_2563508), PE Armenian Hamster anti-mouse CD11c Antibody (1:50 dilution, BioLegend, Cat. #117308, Clone: N418, RRID: AB_313777), PE-eFluor™ 610 anti-mouse CD11B Antibody (1:50 dilution, eBioscience, Cat. #61-0112-80, Clone: M1/70, RRID: AB_2574527), APC Mouse anti-mouse H-2Kb bound to SIINFEKL Antibody (1:50 dilution, BioLegend, Cat. #141605, Clone: 25-D1.16, RRID: AB_11219402), BUV395 Rat anti-mouse CD103 Antibody (1:50 dilution, BD Biosciences, Cat. #740238, Clone: M290, RRID:AB_2739985), Brilliant Violet 711™ Rat anti-mouse CD274 (B7-H1, PD-L1) Antibody (1:50 dilution, BioLegend, Cat. #124319, Clone: 10F.9G2, RRID: AB_2563619). Panel-4.2: Fixable Viability Stain 510 (1:1000 dilution, BD Biosciences, Cat. #564406, RRID: AB_2869572), APC Mouse anti-mouse H-2Kd Antibody (1:50 dilution, BioLegend, Cat. #116620, Clone: SF1-1.1, RRID: AB_10645328) or anti-mouse H-2Kb Antibody (1:50 dilution, BioLedend, Cat. #116518, Clone: AF6-88.5, RRID: AB_10564404), FITC Rat anti-mouse I-A/I-E Antibody (1:50 dilution, BioLegend, Cat. #107606, Clone: M5/114.15.2, RRID: AB_313321), PE/Cyanine7 Mouse anti-mouse CD64 (FcγRI) Antibody (1:50 dilution, BioLegend, Cat. #139314, Clone: X54-5/7.1, RRID: AB_2563904), Brilliant Violet 421™ Rat anti-mouse CD24 Antibody (1:50 dilution, BioLegend, Cat. #101826, Clone: M1/69, RRID: AB_2563508), PE Armenian Hamster anti-mouse CD11c Antibody (1:50 dilution, BioLegend, Cat. #117308, Clone: N418, RRID: AB_313777), PE-eFluor™ 610 anti-mouse CD11B Antibody (1:50 dilution, eBioscience, Cat. #61-0112-80, Clone: M1/70, RRID: AB_2574527), APC Anti-mouse CD86 Antibody (1:50 dilution, BioLegend, Cat. #105012, Clone: GL-1, RRID: AB_493342), BUV395 Rat anti-Mouse CD103 (1:50 dilution, BD Biosciences, Cat. #740238, Clone:M290, RRID: AB_2739985), Brilliant Violet 711™ Dog anti-mouse CD80 Antibody (BioLegend, Cat. #104743, Clone: 16-10A1, RRID: AB_2810338), BUV737 Rat anti-Mouse CD40 Antibody (BD Biosciences, Cat. #741749, Clone:3/23, RRID: AB_2871115). Panel for identification the M1/M2 macrophage phenotypes: Fixable Viability Stain 510 (1:1000 dilution, BD Biosciences, Cat. #564406, RRID: AB_2869572), APC-eFluor™780 Rat anti-mouse CD45

Antibody (1:50 dilution, eBioscience, Cat. #47-0451-82, Clone: 30-F11, RRID: AB_1548781)；FITC Armenian Hamster anti-mouse CD80 Antibody (1:50 dilution, BioLegend, Cat. #104706, Clone: 16-10A1, RRID: AB_313127)；PE/Cyanine7 Rat anti-mouse CD86 Antibody (1:50 dilution, BioLegend, Cat. #105014, Clone: GL-1, RRID: AB_439783)；Brilliant Violet 421™ Rat anti-mouse CD206 (MMR) Antibody (1:50 dilution, BioLegend, Cat. #141717, Clone: C068C2, RRID: AB_2562232)；PE Rat anti-mouse CD163 Antibody (1:50 dilution, BioLegend, Cat. #155308, Clone: S15049I, RRID:AB_2814062)；PerCP-Cy™5.5 Rat anti-mouse CD11b Antibody (1:50 dilution, eBioscience, Cat. #61-0112-80, Clone: M1/70, RRID: AB_2574527)；BUV395 Rat Anti-Mouse F4/80 (1:50 dilution, BD Biosciences, Cat. #565614, Clone: T45-2342, RRID: AB_2739304).
The following antibodies were used for in vivo treatment:the anti-PD-L1 antibody (BioXCell, Cat. #BP0101, Clone: 10F.9G2, RRID: AB_10949073); the isotype control (BioXCell, Cat. #BP0090, Clone: MOPC-21, RRID: AB_1107780); the anti-CD40 antibody (BioXCell, Cat. #BP0016-2, Clone: FGK4.5, RRID: AB_1107601); the anti-CD8β antibody (BioXCell, Cat. #BE0223, Clone: 53-5.8, RRID: AB_2687706); the anti-MHC-I antibody (BioXCell, Cat. #BE0172, Clone: Y-3, RRID: AB_10949300).
The anti-CD45 antibody (1:2000 dilution for IHC and 1:50 dilution for IF, ProteinTech, Cat#60287-1-Ig, Clone:4E9B2, RRID: AB_2881404), anti-FoxP3 (1:200 dilution for IHC and 1:100 dilution for IF, Cell Signaling Technology, Cat#12653, Clone: D6O8R, RRID: AB_2797979), anti-CD8 antibody (1:2000 dilution for IHC, Abcam, Cat#ab209775, Clone: EPR20305, RRID: AB_2860566), anti-CD4 antibody (1:1000 dilution for IHC, Abcam, Cat#ab183685, Clone: EPR19514, RRID: AB_2686917), anti-CD103 antibody (1:1000 dilution for IHC, Abcam, Cat#ab224202, Clone: EPR22590-27, RRID: AB_2936238), anti-CK19 antibody(1:3000 dilution for IHC and 1:100 dilution for IF, ProteinTech, Cat#10712-1-AP, RRID: AB_2133325) and anti-CD31 antibody (1:2000 dilution for IHC, Abcam, Cat#ab182981, Clone: EPR17259, RRID: AB_2920881) were used for IHC- or TSA-based IF- staining.
The anti-CK19 antibody (1:2000 dilution, ProteinTech, Cat#60187-1-Ig, Clone: 3G1E4, RRID: AB_10859834), anti-TNF-α antibody (1:1000 dilution, ProteinTech, Cat#60291-1-Ig, Clone: 7B8A11, RRID: AB_2833255), anti-IFN-γ antibody (1:1000 dilution, Invitrogen, Cat#PA1-24782, RRID: AB_794536), anti-HMGB1 antibody (1:1000 dilution, Abcam, Cat#ab18256, RRID:AB_444360), anti-SpCas9 antibody (1:10000 dilution, Abcam, Cat#ab189380, Clone: EPR18991, RRID:), anti-β-Actin antibody (1:1000 dilution, CST, Cat#3700, Clone: 8H10D10, RRID: AB_2242334), anti-GAPDH antibody (1:5000 dilution, Abcam, Cat#ab9484, Clone: mAbcam9484, RRID: RRID:AB_307274), and anti-PD-L1 antibody (1:2000 dilution, ProteinTech, Cat#66248-1-Ig, Clone: 2B11D11, RRID: AB_2756526) were used for western blotting.

Validation

All commercial antibodies used in the flow cytometry experiment are verified by the company, the detailed information of antibody application and reactivity is provided in Supplementary Table 1. In our study, antibodies were independently validated using the BD LSR Fortessa X-20 instrument before the experiment.

Eukaryotic cell lines

Policy information about cell lines and Sex and Gender in Research

Cell line source(s)

The Panc02 cells were a gift from Dr. Wei Tao (Department of General Surgery, The First Affiliated Hospital, Zhejiang University School of Medicine, Hangzhou, China). The Hepa1-6 cell line (Cat. # SCSP-512）) was bought from the National Collection of Authenticated Cell Cultures (Shanghai, China). The KPC cells were derived from 4 to 6-month-old male KrasLSL-G12D; Tp53fl/+; Pdx1-Cre (KPC) mice. The KL cells were derived from 12 to 16-month old male mice (KrasLSL-G12D; Stk11 fl/fl, AAV9-Cre inhaled).

Authentication

The CK19 markers of pancreatic cancer cells (KPC and Panc02) were validated by IF-staining and western blotting. The mutant KRAS, Trp53 or Stk11(LKB1) allele were validated according to PCR experiments. The Hepa1-6 cell line was authenticated by National Collection of Authenticated Cell Cultures (Shanghai, China).

Mycoplasma contamination

All cells used in the study were tested negative for Mycoplasma contamination.

Commonly misidentified lines
(See ICLAC register)

No commonly misidentified cell lines were used in the study

Animals and other research organisms

Policy information about studies involving animals; ARRIVE guidelines recommended for reporting animal research, and Sex and Gender in Research

Laboratory animals

Male C57BL/6 mice (6-8 weeks old) were purchased from Shanghai Lingchang Laboratory Animal Centre. The KrasLSL-G12D strain (C57BL/6-Krasem4(LSL-G12D) Smoc, RRID: IMSR_NM-KI-190003), Tp53fl/fl strain (C57BL/6-Tp53tm2Smoc, RRID: IMSR_NM-CKO-18005) and Stk11 fl/fl strain (C57BL/6JSmoc-Stk11tm(flox)1Smoc, RRID:IMSR_NM-CKO-18014) were obtained from Shanghai Model Organisms. The mice were kept in the SPF barrier. The facility environment was as follows: Temperature was 20-26 ℃, and daily temperature difference was less than 4℃. Humidity was 40%-60%. The frequency of air change is 15-20 times per hour, and the light and dark alternates every 12 hours. The maximum noise is less than 60dB.

Wild animals

The study did not involve any wild animals.

Reporting on sex

All tumor-bearing mice used in the study were male. This is because male mice are routinely used as models in pancreatic cancer studies that do not involve gender-related factosr. In addition, there is no clear evidence that gender affects the efficacy of pancreatic cancer ablation therapy used in the study.

Field-collected samples

The study did not involve any field-collected samples.

Ethics oversight

The animal experiments were approved by the Committee of Animal Rights and Welfare at Ruijin Hospital Affiliated to Shanghai Jiao Tong University School of Medicine and Committee of Animal Rights and Protection at CAS Center for Excellence in Brain Science and Intelligence Technology, Shanghai.

Note that full information on the approval of the study protocol must also be provided in the manuscript.

Flow Cytometry

Plots

Confirm that:

☒ The axis labels state the marker and fluorochrome used (e.g. CD4-FITC).

☒ The axis scales are clearly visible. Include numbers along axes only for bottom left plot of group (a 'group' is an analysis of identical markers).

☒ All plots are contour plots with outliers or pseudocolor plots.

☒ A numerical value for number of cells or percentage (with statistics) is provided.

Methodology

Sample preparation	The immune cells derived from tumors, TdLNs, spleen, and blood were prepared according to standard protocols. The Hilar lymph nodes, peripancreatic lymph nodes and superior rnesentery lymph nodes were collected from each mouse for subsequent experiments. For cytoplasmic staining of the seecreting proteins IFN-γ,TNF-α or Gzmb, the freshly isolated immune cells were incubated in RPMI-1640 Medium (Sigma, Cat#RNBK0102) containing 10% FBS and 1×Protein Transport Inhibitor Cocktail (Invitrogen, Cat#00-4980-93) for 8 hours in the cell incubator. The Fixable Viability Stain 510 (BD Biosciences, Cat.#564406) were used to discriminate the viable or non-viable cells in the flow cytometry experiments. Next, the total cells were washed using 1×PBS containing 1% BSA, and blocked using CD16/32 antibody(Biolegend, Clone:93, Cat#101320) for 30minutes at room temperature. The cells for cytoplasmic or nuclear staining of IFN-γ, TNF-a, Ki67, and FoxP3 were further processed using intracellular fixation & permeabilizationsets (BD, Cat#554714 or Invitrogen, Cat#225870) according to the manufacturer' protocols. Finally, the cells were incubated in 1×PBS (containing 1% BSA) or permeabilization buffer containing fluorescent-labelled flow cytcometric antibodies, shaking at 4℃ for 12 hours.
Instrument	The flow cytometry experiments were performed using BD LSR Fortessa X-20 instrument.
Software	FlowJo software (version 10.7.1)
Cell population abundance	The abundance of the cell population were calculated using the FlowJo software (version 10.7.1). For certain cell type, the cell counts were firstly calculated using the FlowJo software, and the cell abundance was identified using GraphPad. In each diagram presented in the manuscript, the cell populations involved in the calculation of cell abundance are labeled.
Gating strategy	First, the cell debris are removed according to FSC/SSC gate. Then, the singlets were selected according to FSC-H/FSC-A gate. Subsequently, different populations of cells were selected according to the immunofluorescent features of antibodies and laser wavelength. The gating strategies for different cell populations were presented in the Supplementary Figures.

☒ Tick this box to confirm that a figure exemplifying the gating strategy is provided in the Supplementary Information.
